# Pre- and post-diagnosis costs of tuberculosis to patients on Directly Observed Treatment Short course in districts of southwestern Ethiopia: a longitudinal study

**DOI:** 10.1186/s41043-018-0146-0

**Published:** 2018-05-21

**Authors:** Abyot Asres, Degu Jerene, Wakgari Deressa

**Affiliations:** 1 Department of Public Health, College of Health Sciences, Mizan-Tepi University, PO Box 260, Mizan Aman, Ethiopia; 2 Management Sciences for Health, Addis Ababa, Ethiopia; 3 Department of Preventive Medicine, School of Public Health, College of Health Sciences, Addis Ababa University, Addis Ababa, Ethiopia

**Keywords:** TB, Direct cost, Indirect cost, Longitudinal, Pre-diagnosis, Post-diagnosis, Ethiopia

## Abstract

**Background:**

Financial burden on tuberculosis (TB) patients results in delayed treatment and poor compliance. We assessed pre- and post-diagnosis costs to TB patients.

**Methods:**

A longitudinal study among 735 new TB cases was conducted from January 2015 through June 2016 in 10 woredas (districts) of southwestern Ethiopia. Direct out-of-pocket, payments, and lost income (indirect cost) were solicited from patients during the first 2 months and at the end of treatment. Thus, we ascertained direct medical, nonmedical, and indirect costs incurred by patients during pre- and post-diagnosis periods. We categorized costs incurred from onset of illness until TB diagnosis as pre-diagnosis and that incurred after diagnosis through treatment completion as post-diagnosis. Pre- and post-diagnosis costs constitute total cost incurred by the patients. We fitted linear regression model to identify predictors of cost.

**Results:**

Between onset of illness and anti-TB treatment course, patients incurred a median (inter-quartile range (IQR)) of US$201.48 (136.7–318.94). Of the total cost, the indirect and direct costs respectively constituted 70.6 and 29.4%. TB patients incurred a median (IQR) of US$97.62 (6.43–184.22) and US$93.75 (56.91–141.54) during the pre- and post-diagnosis periods, respectively. Thus, patients incurred 53.6% of the total cost during the pre-diagnosis period. Direct out-of-pocket expenses during the pre- and post-diagnosis periods respectively amount to median (IQR) of US$21.64 (10.23–48.31) and US$35.02 (0–70.04). Patient delay days (*p* < 0.001), provider delay days (*p* < 0.001), number of healthcare facilities visited until TB diagnosis (*p* < 0.001), and TB diagnosis at private facilities (*p* = 0.02) independently predicted increased pre-diagnosis cost. Similarly, rural residence (*p* < 0.001), hospitalization during anti-TB treatment (*p* < 0.001), patient delay days (*p* < 0.001), and provider delay days (*p* < 0.001) predicted increased post-diagnosis costs.

**Conclusion:**

TB patients incur substantial cost for care seeking and treatment despite “free service” for TB. Therefore, promoting early care seeking, decentralizing efficient diagnosis, and treatment services within reach of peoples, and introducing reimbursement system for direct costs can help minimize financial burden to the patient.

**Electronic supplementary material:**

The online version of this article (10.1186/s41043-018-0146-0) contains supplementary material, which is available to authorized users.

## Background

Tuberculosis (TB) remained among the major global public health problems. In 2015, an estimated 10.4 million cases and 1.4 million deaths occurred globally. The African Region constitutes 28% of the global cases and the most severe burden relative to population (281 cases per 100,000 people) [1]. TB morbidity and mortality pose an enormous economic burden to patients, household, and society. Each year, a TB patient loses on average 3 to 4 months of work and up to 30% of household earnings [2].

Efforts to control TB have three distinct, but overlapping humanitarian, public health, and economic dimensions. The efforts imply timely diagnosis and treatment of patients and reduction of costs due to TB [3]. The latest TB control strategy, End TB, underlined the need for universal access to health services without financial hardship, social protection for income replacement, and support in the event of illness [4]. Accordingly, a global target has been set to have no TB-affected family facing catastrophic costs due to TB by 2020 [4, 5]. Costs incurred by a TB patient include either direct or indirect costs. The direct costs comprise out-of-pocket expenses for medical and nonmedical services whereas the indirect costs constitute foregone income because of lost workdays [6].

Despite the free TB diagnosis and treatment, TB patients and families incur high direct and indirect costs due to TB illness [5]. Systematic reviews across low- and middle-income countries showed mean total costs of TB ranging from fewer than I$1 to I$8198 [5, 7]. The review further reported indirect and direct costs incurred for TB care respectively constituted 60 and 40% of the total cost [5]. Studies also reported that seeking care and treatment of TB costs a median of United States of America Dollar (US$) 592 in Nigeria [8] and mean cost of US$108.4 in Yemen [9] per household. The high cost of TB care seeking and treatment result in delays to diagnoses [10, 11] and poor outcome [12, 13]. The poor outcome lead to development of drug resistant TB [14] that require much higher cost of care [15].

Implementation of global TB control strategies in Ethiopia have led to improvements in access to TB care, decline in TB morbidity, and mortality [16]. Nonetheless, Ethiopia belongs to the 14 TB, TB/human immunodeficiency virus (HIV), and multi-drug resistant (MDR) TB high-burden countries [1]. Out of a total US$47.8 million spent for TB control in 2008, household out-of-pocket expenses constituted 62% [17]. A cost and epidemiological modelling in Ethiopia, showed out-of-pocket medical cost for TB amounted to US$49 per patient that led households to fall below poverty line [18]. A study in Tigray, Northern Ethiopia from patient perspective reported median cost incurred for care seeking to be US$16 [19]. A community randomized trial using societal perspective in southern Ethiopia revealed a successful treatment of a smear-positive patient costs US$158.9 at a health facility compared to that within the community (US$61.7) [20].

It is important to understand the financial burden of TB patients to adapt and realize a global target of having no households incurring catastrophic costs because of TB [1]. However, only few studies exist in Ethiopia, those dealt on the financial burden of TB patients. The few studies dealt on cost of care seeking and diagnosis [19] or cost of treatment [20] but not both. The studies were conducted during the 8 months of treatment regimen. However, the current 6-month regimen requires frequent visits (daily to weekly) to health facilities throughout the course of treatment [21]. Furthermore, none of the studies analyzed cost predictors across the continuum of care. Generally, evidences on financial burden posed to TB patients across the pathways to treatment are limited in Ethiopia. Therefore, we studied costs incurred by TB patients across the pre- and post-diagnosis periods including cost drivers in districts of southwestern Ethiopia.

## Methods

### Study setting and design

A longitudinal study among new TB cases on Directly Observed Treatment Short course (DOTS) was carried out from January 2015 through June 2016. We included 14 public healthcare facilities (three hospitals and 11 health centers) from 10 *woredas* (an administrative unit equivalent to district) in three zones (an administrative structure that oversees woredas and report to regional states). The zones, Bench Maji, Kaffa, and Sheka, are among the 15 zones in Southern Nations Nationalities and Peoples Region (SNNPR). The zones are located at the southwestern border of the region where an estimated 2,064,102 people reside [22]. The zones are organized into four town administrations and 26 woredas. During the study, three hospitals and 65 health centers were providing TB DOTS [23].

Diagnosis and treatment for all forms of TB in Ethiopia is according to a national guidelines [21, 24] that specify case definitions, diagnostic, and treatment standards. Diagnosis based on sputum microscopy, sputum follow-up test, and anti-TB drugs are free of charge in all public and selected private facilities. Since the end of 2011, all forms of new TB cases are treated for 6 months with combination of rifampicin (R), isoniazid (H), pyramidal (Z), and ethambutol (E) for the first 2 months (intensive phase) (2RHZE) followed by rifampicin (R) and isoniazid (H) for 4 months (4RH). Thus, patients need to visit a DOTS center daily and weekly during intensive and continuation phase treatments, respectively.

### Sample size and sampling

The sample size needed for the study was calculated using STATA V13 considering a mean (SD) patient cost of care seeking of US$29(14) [19] to detect a US$5 difference which revealed 250 cases. This study is part of a doctoral dissertation aimed at assessing time delays, cost, and outcomes of TB patients on DOTS. Thus, among the sample sizes calculated for each objective of the dissertation, which required for assessing predictors of delay, 802 cases was the largest. Since similar cases were studied for all the objectives, the largest sample, 802 cases was used for this study.

We selected the region (SNNPR) and zones (Bench Maji, Kaffa, and Sheka) conveniently. Considering the available resources and representativeness, we decided to study 10 woredas of the three zones. Thus, through proportional allocation, we determined the number of woredas from each zone. Considering the number of TB cases notified in the preceding year to the study time, we selected the woredas from each zones. Then, all public health facilities providing diagnosis and treatment of TB in the selected woredas were included for the study. We included 14 health facilities (three hospitals and 11 health centers). Therefore, we allocated the 802 cases proportionally to the zones, woredas, and health facilities based on their preceding year TB case notification. Finally, successive consenting cases were enrolled until the required sample reaches. Those new cases, older than 18 years and on intensive phase treatment, were included and those on continuation phase, transferred to other treatment center, lost to follow-up, and died before the enrollment were excluded from study.

### Data collection

Data were collected using structured questionnaire (Additional file 1) adapted from the tool to estimate TB patients’ cost developed by the WHO and other partners [6]. Similarly, data abstraction checklist was prepared to extract data from the standard unit TB register. The questionnaire was translated in to the national language (Amharic) to ease the understanding among both the data collectors and participants. Then, 10 diploma graduate nurse data collectors and three master holder supervisors were recruited and trained for 3 days. The training included the basics of TB control, details of questionnaire, data abstraction, interviewing techniques, and pretest of the tool at health facilities not included in the study. Finally, eligible cases were traced from the unit TB register, and face-to-face interview was held within the intensive phase and end of treatment. The first interview-included patients’ sociodemographic, healthcare-seeking practices, and costs incurred until TB diagnosis (pre-diagnosis cost). The follow-up interview inquired costs incurred after the diagnosis of TB through completion of the treatment (post-diagnosis cost).

### Cost ascertainment

Cost data were ascertained from patient perspective using prevalent approach that estimate financial burden of an illness to patients at specified period of time [25]. So costs incurred by patients for care seeking and treatment of TB were collected at two-time points, during the first 2 months of treatment and at the end of the treatment. Both direct out-of-pocket expenses (for medical and nonmedical services) and indirect costs were measured. Direct costs consisted of out-of-pocket charges for medical services (consultation, drugs, laboratory tests, X-ray, and hospitalization) and nonmedical services (transportation, meal, and accommodation) while visiting healthcare facilities.

Direct out-of-pocket patient expenses during the pre-diagnosis period (incurred from onset of illness to treatment initiation) were determined by asking patient expense at each visit for consultation, laboratory tests, drugs, transportation, meals, and lodging. In the same way, post-diagnosis direct costs (incurred from initiation to completion of the prescribed treatment) were measured by inquiring patients’ medical and nonmedical expenses during visits for anti-TB treatment. The number of visits for the pre-diagnosis period was solicited from patients, and post-diagnosis visits were taken from attendance records on a unit register. Thus, transportation cost was calculated by multiplying the number of visits with the fee per trip.

The indirect costs were estimated using the human capital approach. Patients were requested to estimate time lost due to sickness and visits for consultation, hospitalization, drug collection, and trip journey. The time spent second or minute were converted to hours and then to days at an average of eight working hours in a day (8 h = 1 day). Finally, the number of days was multiplied by an average daily wage rate of US$2.43 = 50 Ethiopian Birr for those unemployed and daily rate calculated from their gross monthly salary for those formally employed. Monthly income of formally employed cases was inquired from patients by asking their monthly salary. For self-employed, self-estimated average monthly earning was used to calculate daily rate. An average wage rate estimated by the social affair offices was used for unemployed cases.

For cost items with no charge, it was recorded as zero. All the costs were inquired in local currency, Ethiopian Birr (ETB), and then converted into US dollars (US$) using the average exchange rate of (US$1 = 20.56 ETB) during January through December 2015 [26].

### Data management

Data were entered into EpiData v3.1 then exported to SPSS version 21 for cleaning and then to STATA 13 for analysis. The data were described using frequency, proportions, mean (standard deviation), and median (inter-quartile range), and normality of the cost data were checked using plots (Q-Q plots and/or histograms) or Kolmogorov-Smirnov test. The cost data were right skewed and became log normal upon log transformation to base 10. Hence, all the statistical tests were done with the lognormal data and reported by back transforming to its anti-log.

Proportion and mean differences across categorical variables were tested using chi-square and independent *t* tests respectively. Associations between continuous variables were tested with simple correlation. Mean difference between pre- and post-diagnosis costs were tested with paired *t* test. Finally, simple and multiple linear regression models were fitted to identify predictors of pre- and post-TB diagnosis costs. Assumptions and fitness for the linear regression model were assessed and ensured. In all the statistical tests, significance was judged at *p* < 0.05.

### Ethical issues

The Institutional Review Board of the College of Health Sciences at Addis Ababa University approved the study protocol. Therefore, patients consented in written for the interview and clinical records of patients were retrieved upon permission from the respective health facilities.

### Operational definitions

*Patient delay* is days elapsed between onsets of illness to the first formal healthcare seeking.

*Health system/provider delay* is the number of days spent between the first consultations to initiation of treatments.

*Total delay* is the number of days elapsed since onset of illness to anti-TB treatment initiation.

*Medical cost* is costs incurred for medical services including consultation, laboratory tests, drugs other than anti-TB, X-ray, and related services.

*Nonmedical cost* is costs incurred for transportation, accommodation, meal, and related services while seeking care for TB and visiting to collect anti-TB drugs.

*Direct cost* is out-of-pocket patient expenses for medical or nonmedical services while seeking care, diagnosis, and treatment for TB.

*Indirect cost* is lost earning because of inability to work or lost workdays while traveling to seek care, diagnosis, and treatment for TB.

*Pre-diagnosis cost* is the cost incurred since onset of illness until anti-TB treatment initiation.

*Post-diagnosis cost* is the cost incurred since the beginning up to the completion of anti-TB treatment.

*Total cost* is both direct and indirect costs incurred for care seeking, diagnosis, and treatment of TB.

## Results

A total of 735 TB cases were enrolled of which 627(85.3%) completed the follow-up. Those lost included 29(3.9%) deaths, 36(4.9%) transferred to other treatment centers, 5(0.7%) treatment failure, and 38(5.2%) lost to follow-up. Nonetheless, there were no statistical significant differences with the proportions of the attributes across the baseline and end line surveys (Table 1). The median age (inter-quartile range (IQR)) of the cases during enrollment (baseline) was 27(20–37) years. Of the cases enrolled, 53 and 29.4% completed elementary school and are involved in farming, respectively. The mean (+ SD) of size and median annual income of the households were 4.3(+ 2.1) and US$466.93, respectively.

**Table 1:** Sociodemographic characteristics of TB cases in districts of southwestern Ethiopia, January to December 2015

Variable		Baseline (*n* = 735) *n*(%)	End line (*n* = 627) *n*(%)	*P* value
Gender	Female	288(39.2)	244(38.9)	0.9
Age(years)	18–34	503(68.4)	431(68.7)	0.9
	35–65	216 (29.4)	183(29.1)	0.8
	> 65	16(2.2)	13(2.1)	0.89
Marital status	Never married	275(37.4)	235(37.5)	0.97
	Currently married	404(55)	342(54.5)	0.85
	Widowed/divorced	56(7.6)	50(8)	0.78
Educational status	No formal education	212(28.8)	176(28.1)	0.77
	Completed elementary	389(53)	340(54.2)	0.66
	Secondary and above	134(18.2)	111(17.7)	0.8
Occupation	Employed	172(23.4)	147(23.4)	1.00
	Farming	216(29.4)	188(30.0)	0.81
	Unskilled work^a^	51(6.9)	43(6.9)	1.00
	Dependents^b^	296(40.3)	249(39.7)	0.82
Residence	Urban	369(50.2)	313(49.9)	0.94
	Rural	367(49.9)	314(50.1)	0.74
Household main income earner	Self	370(50.3)	310(49.4)	0.74
	Other^c^	365(49.7)	317(50.6)	0.8
Household income	≤ US$466.93	288(50.6)	242(50.3)	0.9
	> US$466.93	281(49.4)	239(49.7)	0.9

^a^Housemaid, daily laborer

^b^Students, housewife

^c^Father/mother/husband/wife/brother/sister/employer

### Care-seeking pathways

TB patients first visited a healthcare facility after a median of 25 days from onset of illness (patient delay). Thus, 35.4 and 32.4% of the cases first visited private clinics and public health centers, respectively (Table 2). The rest of the cases first visited hospitals (30.1%) and health posts (2.1%). TB diagnosis of 448(61%) cases was made at a hospital, and for 244(33.2%), the diagnosis was made at the first visited health facility. Diagnosis of 491(66.8%) were reached after an average (+SD) of 3.6(+ 2.4) visits to an average (+SD) of 2.2(+ 1.2) healthcare facilities (HCF). Since the first consultation, a median of 22 days had been elapsed to initiate anti-TB treatment. Among the cases, 586(79.7%) had pulmonary TB and 362(49.3%) were diagnosed clinically. All of the cases were offered HIV screening test of whom 68 (9.3%) tested positive, 95% CI (7.2–11.3%).

**Table 2:** Care-seeking pathways and clinical characteristics of TB cases on treatment in districts of southwestern Ethiopia, January to December 2015

Variable		Baseline (*n* = 735)	End line (*n* = 627)	*P* value
		*n*(%)	*n*(%)	
Action to illness before visiting HCF	None	586(79.7)	497(79.3)	0.85
	Took action^a^	149(20.3)	130(20.7)	0.85
First visited HCF	DOTS center	459(62.5)	387(61.7)	0.85
	Non-DOTS	276 (37.5)	240(38.3)	0.76
Diagnosis made HCF	Public	636(86.5)	537(85.6)	0.76
	Private	99(13.5)	90(14.4)	0.70
Type of TB	Pulmonary positive	373(50.7)	320(51.0))	0.90
	Pulmonary negative	213(29.0)	176(28.1)	0.70
	Extra pulmonary	149(20.3)	131(20.9)	0.70
Mode of diagnosis	Bacteriological	373(50.7)	319(50.9)	0.90
	Clinical	362(49.3)	308(49.1)	0.90
Treatment center	Health center	469(36.2)	408(65.1)	0.70
	Hospital	266(63.8)	219(34.9)	0.70
Travel time to treatment center	≤ 1 h	437(59.5)	373(59.5)	1.00
	> 1 h	298(40.5)	254(40.5)	1.00
Hospitalized for treatment	Yes	19(2.6)	15(2.4)	0.40
HIV co-infection	Yes	68(9.3)	46(7.3)	0.10
Patient delay	Median (IQR) days	25((15–36)	23(14–34)	0.20
Provider delay	Median (IQR) days	22(9–48)	20(8–48)	0.40
Total delay	Median (IQR) days	55(32–100)	52(31–93)	0.50

^a^Self-treatment, consult traditional healer, used holy water

### Pre-diagnosis cost

**Fig. 1:**
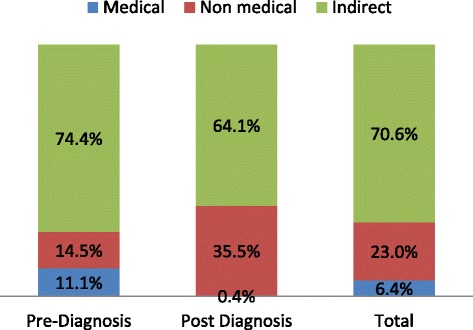
Distribution of TB patient costs across pre and post diagnosis periods in districts of southwestern ethiopia January 2015 to June 2016

Until diagnosis of TB, patients incurred a median (IQR) cost of US$97.6 (56.4–184.2) (Table 3). Direct cost amount to median (IQR) US$21.64 (10.23–48.31) and constitute 25.6% (Fig. 1) of the total pre-diagnosis costs. Patients had lost median (IQR) of 24.7(15.1–48.4) workdays until diagnosis of TB that corresponded to median (IQR) US$64.45 (39.8–128.8) income loss.

**Table 3:** Distribution of TB patient costs across cost categories and periods in districts of southwestern Ethiopia, January to December 2015

Cost category		Cost period		
		Pre-diagnosis (US$)	Post-diagnosis (US$)	Total (US$)
Medical	Mean (95% CI)	8.56 (7.68, 9.54)	4.4 (3.23, 6.0)	8.75 (7.85, 9.75)
	Median (IQR)	10.72 (4.58, 23.76)	0 (0)	11.19 (4.73–24.08)
Nonmedical	Mean (95% CI)	10.08 (8.99, 11.30)	43.27 (38.32, 48.23))	64.1 (58.34, 69.9)
	Median (IQR)	8.27 (1.61, 24.32)	35.02 (0–70.04)	37.11 (14.35, 85.12)
Total direct	Mean (95% CI)	21.46 (19.65, 23.43)	43.80 (38.82, 48.78)	84.82 (77.92, 91.72)
	Median (IQR)	21.64 (10.23, 48.31)	35.02 (0–70.04)	59.58 (29.43, 113.81)
Indirect	Mean (95% CI)	75.62 (70.68, 80.90)	75.20 (69.14, 81.26))	140.31 (132.35, 148.74)
	Median (IQR)	64.45 (39.82, 128.80)	51.07 (34.65, 93.02)	127.68 (78.43, 201.85)
Total	Mean (95% CI)	108.0 (101.31, 115.11)	117.0 (110.47, 123.87)	244.71 (229.45, 260.98)
	Median (IQR)	97.62 (56.43, 184.22)	93.75 (56.91, 141.54)	201.48 (136.7, 318.94)

The pre-diagnosis cost was positively correlated with patient (*γ* = 0.32, *p* < 0.001), provider (*γ* = 0.64, *p* < 0.001) and total delays (*γ* = 0.68, *p* < 0.001), and the number of HCF visited until diagnosis (*γ* = 0.42, *p* < 0.001). The mean pre-diagnosis cost was significantly different across the types of TB (F = 10.03, *p* < 0.00), type of first visited HCF (*p* = 0.002), HCF where diagnosis was made (*p* < 0.001), and mode of diagnosis (*p* = 0.001) (Additional file 2: Table S1).

In a multiple regression patient and provider delays, being clinically diagnosed, TB diagnosis at private facilities and the number of visited healthcare facilities independently predicted higher mean pre-diagnosis costs (Table 4). Every single patient and provider delay days each increased the mean pre-diagnosis cost by 0.5%. Those patients’ diagnosed clinically incurred 11% higher mean pre-diagnosis costs compared to those diagnosed bacteriologically. Similarly, patients diagnosed at private HCFs incurred 18% higher mean pre-diagnosis cost compared to those diagnosed at public HCFs.

**Table 4:** Predictors of pre-diagnosis cost among TB cases on treatment in districts of southwestern Ethiopia January to December 2015

Variable		Mean(SD) (US$)	Unadjusted exp.^a^coefficient (95% CI)	*P* value	Adjusted exp.^b^coefficient (95% CI)	*P* value
Gender	Male	112.62(0.12)	Ref.		Ref.	
	Female	101.25(0.11)	0.90(0.79, 1.02)	0.1	0.94(0.86, 1.03)	0.20
HIV result	Positive	131.2(0.13)	1.24(0.98, 1.55)	0.07	1.16(0.99, 1.36)	0.06
	Negative	106.02(0.11)	Ref.		Ref.	
Mode of diagnosis	Bacteriological	97.00(0.11)	Ref.		Ref.	
	Clinical	121.08(0.12)	1.25(1.10, 1.42)	0.001	1.08(0.99, 1.18)	0.09
Residence	Urban	114.61(0.12)	Ref.		Ref.	
	Rural	102.13(0.1)	0.89(0.78, 1.01)	0.07		
Patient delay^c^(days)			1.006(1.005, 1.006)	< 0.001	1.005(1.004, 1.01)	< 0.001*
Provider delay^c^(days)			1.006(1.005, 1.01)	< 0.001	1.005(1.004, 1.01)	< 0.001*
Action before HCF visit	None	106.72(0.12)	Ref.		Ref.	
	Took action^d^	113.41(0.11)	1.23(1.07, 1.41)	0.03	1.15(1.03, 1.28)	0.01
First visited HCF	DOTS center	99.97(0.11)	Ref.		Ref.	
	Non-DOTS center	122.57(0.11)	1.23(1.07, 1.40)	0.002	1.01(0.91, 1.11)	0.8
TB diagnosed HCF	Public	101.78(0.11)	Ref.		Ref.	
	Private	153.88(0.13)	1.5(1.26, 1.81)	< 0.001	1.18(1.03, 1.34)	0.02*
Number of HCF visited until diagnosis^b^			1.40(1.34, 1.47)	< 0.001	1.18(1.13, 1.23)	< 0.001*

*Statistically significant at *p* < 0.05

^a^Exponent to the power of 10

^b^Adjusted for all the variables listed in the table

^c^Variable treated as continuous

^d^Self-treatment, used holy water, consult traditional healer

### Post-diagnosis cost

After the diagnosis of TB, patients incurred a total median (IQR) of US$93.75 (56.9–141.54) until the completion of the treatment (Table 3). The direct cost amounted to a median (IQR) of US$35.02 (0–70.04) and constitutes 35.9% (Fig. 1) of the total post-diagnosis cost. During the treatment, TB patients had lost a median (IQR) of 21(13–35.3) workdays that corresponded to a median (IQR) of US$51.0 (34.6–97.0) income loss (indirect cost). Thus, significantly lower medical and indirect costs and higher nonmedical costs were incurred during the post-diagnosis period compared to the pre-diagnosis. The post-diagnosis cost was positively correlated with patient (*γ* = 0.20, *p* < 0.001) and provider (*γ* = 0.23, *p* < 0.001) delays.

In the multiple regression analysis, being a rural resident, having a travel time beyond 1 h to the treatment center, being admitted for anti-TB treatment, patient and provider delays independently predicted higher mean post-diagnosis cost. On the other hand, completing primary and higher educational status and being treated at a hospital predicted lower mean post-diagnosis costs (Table 5). Thus, being a rural resident was associated with an increase in mean post-diagnosis cost by 48% compared to those being urban residents. Every patient and provider delay days increases the mean post-diagnosis cost by 0.3 and 0.2%, respectively. Those patients hospitalized for anti-TB treatment had more than twofold higher mean post-diagnosis cost compared to those patients never hospitalized for anti-TB treatment. Patients who followed their course of anti-TB treatment at hospitals had about 18% lower mean post-diagnosis cost compared to those who received the anti-TB treatment at health centers.

**Table 5:** Predictors of post-diagnosis cost among TB cases on treatment in districts of southwestern Ethiopia January to December 2015

Variable		Mean(SD)	Unadjusted exp^a^coefficient (95% CI)	*P* value	Adjusted exp^b^coefficient (95% CI)	*P* value
Gender	Male	119.73(0.1)	Ref.		Ref.	
	Female	112.51(0.1)	0.94(0.83, 1.06)	0.3	0.92(0.83, 1.02)	0.07
Residence	Urban	94.02(0.11)				
	Rural	154.7(0.08)	1.64(1.48, 1.83)	< 0.001	1.48(1.34, 1.64)	< 0.001*
Educational status	Illiterate	137.15(0.09)	Ref		Ref	
	Primary	117.35(0.09)	0.86(0.74, 0.98)	0.03	0.87(0.77, 0.97)	0.02*
	Secondary and above	97.79(0.08)	0.71(0.60, 0.84)	< 0.001	0.83(0.72, 0.95)	0.01*
HIV result	Positive	123.52(0.1)	1.06(0.88, 1.28)	0.5	1.13(0.97, 1.30)	0.1
	Negative	116.20(0.1)	Ref.		Ref.	
Mode of diagnosis	Bacteriological	118.25(0.1)	Ref.		Ref.	
	Clinical	115.82(0.1)	0.98(0.87, 1.10)	0.7	0.96(0.87, 1.05)	0.3
Treatment center	Hospital	103.05(0.1)	0.78(0.70, 0.88)	< 0.001	0.82(0.74, 0.90)	< 0.001*
	Health center	131.43(0.1)				
Travel time to treatment center	> 1 h	105.7(0.08)	1.37(1.22, 1.55)	< 0.001	1.09(1.02, 1.21)	0.03*
	≤ 1 h	145.14(0.09)	Ref.		Ref.	
Patient delay^c^			1.003(1.002, 1.004)	< 0.001	1.003(1.001, 1.003)	< 0.001*
Provider delay^c^			1.002(1.001, 1.003)	< 0.001	1.002(1.001, 1.002)	< 0.001*
Action before HCF visit	None	111.85(0.11)	Ref.		Ref.	
	Took action^d^	137.34(0.1)	1.23(1.07, 1.41)	0.003	1.08(0.98, 1.28)	0.1
TB diagnosed HCF	Public	116.37(0.1)	Ref.			
	Private	121.27(0.1)	1.04(0.87, 1.24)	0.5	0.95(0.83, 1.09)	0.5
Hospitalized for treatment	Yes	240.82(0.1)	2.13(1.61, 2.80)	< 0.001	2.3(1.84, 2.88)	
						< 0.001*
	No	113.3(0.1)	Ref.		Ref.	

*Statistically significant at *p* < 0.05

^a^Exponent to the power of 10

^b^Adjusted for variables in the table

^c^Variable treated as continuous

^d^Self-treatment, used holy water, consult traditional healer

### Total cost of TB care seeking and treatment

Total costs incurred by patients for care seeking, diagnosis, and treatment amount to a median (IQR) of US$201.48 (136.70–318.94) (Table 3). Pre- and post-diagnosis costs respectively constituted 53.6 and 46.4% of the total cost. Total direct cost constituted 29.4% (Fig. 1) of the total cost and amounted to a median (IQR) of US$59.58 (29.43–113.81). Drugs other than anti-TB and diagnostic tests (laboratory or imaging tests) corresponded to 49.7 and 44.6% of the total medical costs, respectively. During the care seeking and treatment visits, patients had totally lost a median (IQR) 51.7 (32.0–80.8) workdays that corresponded to a median (IQR) of US$127.68 (78.43–201.85) income loss (indirect cost). Out of the total forgone income due to the TB illness, the loss due to lost workdays following care-seeking visits amounted to a median (IQR) of US$18.02 (11.35–30.85) that constitutes 28.4% of the total indirect cost. For 471/569 (82.8%) of the cases, the total cost represents more than 10% of their estimated household annual income.

The mean total cost is significantly different across the types of TB (F = 3.68, *p* = 0.03), action taken before HCF visit (*p* = 0.01), travel time to treatment center (*p* = 0.001), and hospitalization for anti-TB treatment (*p* = 0.005) (Additional file 2: Table S1). In multiple regression, rural residence, travel time to treatment center beyond 1 h, action taken before HCF visit, hospitalized for anti-TB treatment, number of visited HCF, and patient and provider delays all independently predicted increased mean total patient cost of TB care (Additional file 2: Table S2). The mean total cost incurred by patients who are rural residents is about 24% higher than that by urban residents, adjusted exp. coefficient (AeC) (95% CI) 1.24 (1.13, 1.4). Similarly, every patient and provider delay day predicts about 0.3% each AeC (95% CI) 1.003(1.002–1.004) increment in mean total patient cost. Those patients who took action before initiating HCF visits had incurred 17% higher mean total cost compared to those who did not took action. Hospitalization during anti-TB treatment increase the total mean patient cost by 97% compared to those not hospitalized.

## Discussion

This follow-up study of new TB cases on DOTS revealed patients incurred substantial cost across pathways to TB treatment. Thus, the median out-of-pocket payment for an episode of TB illness amounted to US$59.58 that constitutes more than a quarter (29.4%) of the total cost. More than half (53.6%) of the total cost were incurred before diagnosis of TB, and majority (70.6%) of the total cost were attributed to nearly 52 lost workdays per patient. Compared to the post-diagnosis, patients incurred significantly higher medical and indirect costs and lower nonmedical costs during the pre-diagnosis period. Increased pre-diagnosis costs were attributed to patient and provider delays, taking informal treatment before HCF visit, diagnosis at private facilities, being clinically diagnosed, and the number of visited health facilities. On the other hand, rural residence, hospitalization for anti-TB treatment, and following anti-TB treatment at a health center predicted increased post-diagnosis patient costs.

The total cost incurred across the care-seeking and treatment pathways are significantly correlated with both patient and provider delays. The increased pre-diagnosis cost with patient delay could be due to increased risks of severe manifestation [27] that lead to hospitalization and companion during care seeking and treatment. Besides, the patient delay is associated with informal care including self-treatment and traditional cares [28, 29] that pose costs to patients. The patient delays are accompanied by longer lost workdays that reduced patient income. On the other hand, the delay at health system (provider delay) is associated with repeated visits to different HCF when patients incur for both medical and nonmedical services.

Those patients diagnosed clinically incur significantly higher pre-diagnosis cost. This could be due to national diagnostic algorithm that respectively requires 2–4 and 4–8 weeks follow-up for the clinical diagnosis of smear negative and extra pulmonary TB [21]. The higher costs for the clinical diagnosis could be due to the requirement of experienced clinician decisions guided by better diagnostic facilities. Such experts and facilities exist at only few healthcare facilities situated in cities very far from the majority of the people that lead to higher transportation and lodging costs to patients. The repeated consultations and diagnostic tests until diagnosis of TB all incur cost to the patients. The relatively lower cost of bacteriologically confirmed diagnosis could be due to exemption of sputum smear microscopy and culture by the national TB control program. Hence, ensuring efficient diagnostic algorithms and quality bacteriological tests can reduce the financial burden of TB patients.

Consistent with other studies [8, 30] patients diagnosed at private facilities incur significantly higher pre-diagnosis cost compared to those diagnosed at public facilities. The different cost items and rates at the private facilities where every services including sputum microscopy is charged can explain the relatively higher cost at the private. Since there were no public-private mix (PPM)-DOTS in the study area at the time of study, the private HCF might not implement the proper diagnostic algorithm that might lead to delay and extra cost. Furthermore, public health facilities requirement of retesting a positive sputum result from private facilities for treatment initiation leads to delay and extra cost [31].

We found patients treated at hospitals had significantly lower post-diagnosis cost compared to those treated at health center. This could be due to the presence of full-time staff that exclusively provides TB patient care at hospitals. However, at health centers, providers are given multiple duties other than TB DOTS that increase patient waiting time and costs. In addition, health centers are situated in rural areas where there is no transport access within villages in contrast to hospitals in urban areas easily accessible to patients within the town. Thus, statistically significant difference in mean total time (71.17 vs 106.79 min, *p* < 0.001) had been respectively spent per each patient visit to hospitals and health centers. Consistent with other studies [8, 32], we found significantly higher post-diagnosis cost incurred by patients from rural areas compared to those from urban areas. The reason could be due to significantly higher mean time spent per each visit among patients that are rural and urban residents (119.51 vs. 68.35 min, *p* < 0.001), respectively.

Our study had some limitations. First, cost measurements relied on patient recall, which was liable to recall bias. However, we did the baseline interview within the first 2 months of treatment when patients are highly likely to recall about the costs they incurred. Second, the study employed only patient perspective so that we were not able to determine costs incurred by health systems, households, and communities. Lastly, we determined the cost based on a prevalent approach that measure costs for an episode of illness so that we could not determine lifetime cost of TB illness. On the other hand, our study employed a longitudinal design involving a relatively large sample recruited consecutively. As a result, selection bias was minimized and patient costs from care seeking through treatment completion were determined. The findings in the paper are valid but need to be interpreted cautiously considering the limitations. Given the internal validity, the findings can be applied to patients in similar settings since the characteristics of patients and the health systems in similar settings might not differ significantly.

## Conclusion

The study revealed TB patients on DOTS incur substantial cost across the pathways to anti-TB treatment despite the “free service.” Significantly, higher cost was incurred during the pre-diagnosis period compared to the post-diagnosis period showing longer pathways of care seeking. Increased pre-diagnosis costs are attributed to patient and provider delays, informal care before consultation, seeking care at private healthcare facilities, and clinical diagnosis. Higher post-diagnosis costs are attributed to patient and provider delays, rural residence, and being treated at health center. Thus, implementation of patient-centered TB care introducing reimbursement mechanisms and scaling up of national community and social insurance initiatives to the study area are vital to reduce patients’ out-of-pocket expenditures. In addition, introducing reimbursement of direct costs, promoting early care seeking, equipping healthcare facilities with the necessary equipments, and staffing with qualified health work force, and decentralizing efficient diagnosis and treatment within reach of patients can minimize the patient costs.

## Additional files

Additional file 1: Consent form and questionnaire. (DOCX 112 kb)

Additional file 2: **Table S1.** Mean differences of pre, post, and total costs to patients among TB cases on treatment in districts of southwestern Ethiopia January to December 2015. Table S2. Predictors of total cost to patients among TB cases on treatment in districts of southwestern Ethiopia January to December 2015. (DOCX 25 kb)

## Abbreviations

CI: Confidence interval
DOTS: Directly Observed Treatment Short course
ETB: Ethiopian Birr
HCF: Healthcare facility
HIV: Human immunodeficiency virus
IQR: Inter-quartile range
PPM: Public-private mix
SD: Standard deviation
TB: Tuberculosis
US$: United States of America Dollar
