# Supplementary material for: Pre- and post-diagnosis costs of tuberculosis to patients on Directly Observed Treatment Short course in districts of southwestern Ethiopia: a longitudinal study
Source: J Health Popul Nutr. 2018 May 21;37:15. doi: 10.1186/s41043-018-0146-0 (PMC5963051; doi:10.1186/s41043-018-0146-0)
Supplement: Supplementary file 1 — Consent form and questionnaire. (DOCX 112 kb) [file 41043_2018_146_MOESM1_ESM.docx]

**Additional file 1: Consent Form**

My name is ____________________________. I am working with a research team from Addis Ababa University. The purpose of the study is to assess delays in care seeking and treatment for TB, the cost incurred for the care seeking that is helpful to design interventions for the delays and financial burden posed by TB. We are interviewing systematically selected TB patients on treatment about their care seeking practices, treatment practices, and cost incurred for care seeking and treatment. I am going to ask you some questions regarding your sociodemographics, care seeking practices, and cost incurred for the care seeking and treatment that are crucial for the proposed research. Thus, your honest responses will enable design and implementation of interventions that enhance control of the TB disease. Your responses will be completely confidential which will be ensured by avoiding your name or other identifications in connection with any of the information you tell me. You do not have to answer any question that you do not want to answer, and you may end this interview at any time you wish. We would greatly appreciate your help in responding to this survey.

Person to contact:

**Principal Investigator:**

*Abyot Asres* (Mizan Tepi University, *Mob: +251911905554* E-mail: abyotas@yahoo.com)

**Supervisors**: Dr. Wakgari Deressa (School of Public Health, College of Health Science, Addis Ababa University, Cell phone: +251 911483714) and;

Dr. Degu Jerene (Cell phone: +251 911546407)

1. Are the information/ objectives clear?

1. Yes 2. No explain again

1. Would you be willing to participate?
2. Yes, Signature ________________________ continue the interview
3. No Stop and go to the next patient

Thank him/her

1. Interviewer’s signature certifying that, the informed consent has been given by the respondent

Name____________________ Signature _____________ Date_____________

**Baseline English version questionnaire**

- 1. General information about study area

| Questionnaire ID |  |  | |  | |  |  |  | |
| --- | --- | --- | --- | --- | --- | --- | --- | --- | --- |
| Name of Zone/ | 1. Kaffa | | 1. Bench Maji | | | | | | 1. Sheka |
| Name of woreda | 1. Bonga town 2. Decha 3. Chena | | 1. Mizan Aman 2. Shey Bench 3. North Bench 4. Meinet Goldiya | | | | | | 1. Tepi town 2. Yeki 3. Masha |
| Name of Health Facility |  | | | | | | | | |
| Type of Health facility | 1. Hospital 2. Health center 3. Health post 4. Other (specify)------ | | | | | | | | |
| Interviewee | 1. Patient 2. Treatment supporter 3. Other specify------ | | | | | | | | |
| Date of interview |  | | | | Follow-up visit schedule: | | | | |
| Name and signature of interviewer |  | | | | Name and signature of supervisor: | | | | |

| - 1. **Medical record review checklist** | | | | | | | | | | |
| --- | --- | --- | --- | --- | --- | --- | --- | --- | --- | --- |
| Medical record number | _________________ | | | Date of visit (s):________________________ | | | | | | |
|  |  |  |  | Duration of illness (dd,week,mm) | | | | | | |
| Chief complaint | 1. Cough | | |  | | | | | | |
|  | 1. Fever | | |  | | | | | | |
|  | 1. Chest pain | | |  | | | | | | |
|  | 1. Night sweating | | |  | | | | | | |
|  | 1. Swelling over neck | | |  | | | | | | |
|  | 1. Other specify----------------------- | | |  | | | | | | |
| Key physical findings | 1. HEENT | | |  | | | | | | |
|  | 1. LGS | | |  | | | | | | |
|  | 1. Chest | | |  | | | | | | |
|  | 1. CVS | | |  | | | | | | |
|  | 1. Abdomen | | |  | | | | | | |
|  | 1. Other specify------------------------- | | |  | | | | | | |
| Investigations requested ,their results and unit cost | Investigation | | | Result | | Unit cost of the investigation | | | | |
|  | 1. Sputum for AFB | | |  | |  | | | | |
|  | 1. Xpert MTB/RIF | | |  | |  | | | | |
|  | 1. CXR(chest x ray) | | |  | |  | | | | |
|  | 1. ESR | | |  | |  | | | | |
|  | 1. WBC | | |  | |  | | | | |
|  | 1. Other specify | | |  | |  | | | | |
| Diagnosis/Assessment | Diagnosis | | | Date of diagnosis | | | | | | |
|  | 1. TB | | |  | | | | | | |
|  | 1. Pneumonia | | |  | | | | | | |
|  | 1. B.Asthma | | |  | | | | | | |
|  | 1. Other specify------------------------- | | |  | | | | | | |
|  | 1. Not stated/written | | |  | | | | | | |
| Treatments/plan prescribed and their unit cost |  | | | Unit cost of the treatment prescribed | | | | | | |
|  |  |  |  |  | | | | | | |
| - 1. **Unit TB register review** | | | | | | | | | | |
| TB unit number | |  | **Woreda** | |  | | | | **kebele** |  |
| Sex | | 1. Male 2. Female | | | | | | | | |
| Age | | ________________years | | | | | | | | |
| Smear result | | 1. Positive 2. Negative 3. Not done | | | | | | **Lab number**:__________ | | |
| Baseline/intial weight (Kg) | | _________________ | | | | | | | | |
| Patient category | | 1. New 2. Transfer in 3. Other 4. Other specify____________ | | | | | | | | |
| Type of TB | | 1. PPos 2. Pneg 3. EPTB | | | | | | | | |
| Intensive drugs | | 1. RHZE 2. SRHZE 3. Other ----- | | | | | Dose (#of tabs):________ | | | |
| Treatment started date/dd/mm/yy | | _____________________ | | | | | | | | |
| HIV test offered | | 1. Yes 2. No | | | | | | | | |
| HIV test performed | | 1. Yes 2. No | | | | | | | | |
| HIV result | | 1. Reactive 2. Non-reactive 3. NA | | | | | | | | |
| CPT started date | | 1. Yes 2. No if yes when-----------------dd/mm/yy | | | | | | | | |
| Date HIV care enrolled | | Yes 2. No if yes when-----------------dd/mm/yy | | | | | | | | |
| Date ART started | | Yes 2. No if yes when-----------------dd/mm/yy | | | | | | | | |
| ART unique number | |  | | | | | | | | |

Part I; Patients’ general background information (socidemographic and Socioeconomic characteristics)

| Q no | Questions | Options/responses | | | Skip |  |
| --- | --- | --- | --- | --- | --- | --- |
| 101 | Your usual residence | 1. Urban 2. Rural (woreda-----kebele---- | | |  |  |
| 102 | Your current marital status | 1. Never married 2. Married 3. Widowed 4. Divorced/Separated | | |  |  |
| 103 | To which religion do you belong? | 1. Orthodox Christian 2. Muslim 3. Catholic 4. Protestant Christian 5. Traditional 6. Other specify-------- | | |  |  |
| 104 | To which ethnic group do you belong? | 1. Kafficho 2. Bench 3. Shekacho 4. Sheko 5. Amhara 6. Tigire 7. Oromo 8. Meinit 9. Other (specify | | |  |  |
| 105 | What is your highest level of education completed? | 1. Never attended formal school 2. 1-4 (First cycle primary 3. 5-8 (2^nd^ cycle primary) 4. 9-10/12 5. Graduate/certificate (10+1/2/3 or 12+1/2/3/4) 6. Other (specify-------- | | |  |  |
| 106 | What is your main occupation? | 1. Government employee 2. Private employee 3. Self employed 4. Housemaid 5. House wife 6. Student 7. Farmer 8. Daily laborer 9. Merchant 10. Other (specify-- | | |  |  |
| **Part II. Care seeking practices and costs incurred for the illness until diagnosis of TB** | | | | |  |  |
| 201 | When was the current illness you are being treated started? | | 1. -------/---------/-------dd/mm/yy 2. I donot know/remember | |  |  |
| 202 | What was/were your chief symptoms and date of onset of the current illness?  **Note for interviewer: More than one response possible and check for that without reading options** | | 1. Cough (dd/mm/yy): [ ] [ ] [ ] 2. Fever (dd/mm/yy): [ ] [ ] [ ] 3. Loss of Weight:(dd/mm/yy): [ ] [ ] [ ] 4. Haemoptysis (dd/mm/yy): [ ] [ ] [ ] 5. Chest pain : (dd/mm/yy): [ ] [ ] [ ] 6. Others (specify) (dd/mm/yy): [ ] [ ] [ ] | |  |  |
| 203 | Which symptom(s) made you seek care?  **Note for interviewer: More than one response possible and check for that without reading options** | | 1. Cough 2. Fever 3. Loss of Weight 4. Haemoptysis 5. Chest pain 6. Others (specify-------------------------------------- | |  |  |
| 204 | Where and when did you first seek care for the complaints? | | **Action taken** | **Date of visit/action** | 206 | |
|  |  |  | 1. Self medication |  |  |  |
|  |  |  | 1. Used traditional medicine |  |  |  |
|  |  |  | 1. Used holly water |  |  |  |
|  |  |  | 1. Consult HEW |  |  |  |
|  |  |  | 1. Consult HCP at HF |  |  |  |
|  |  |  | 1. Other (specify--------------) |  |  |  |
| 205 | Why did not you consult health facility first during the onset of symptoms?  **Note for interviewer: Health facility is to mean private clinic or hospital, public health center, or hospital or health post**  **More than one response possible and check for that without reading options** | | 1. Thought it was mild and relived by it self 2. Health facilities were too far 3. Facility do not provide the service 4. Too busy/long waiting time 5. Previous bad experiences 6. Fear of being diagnosed for TB 7. TB is common and cure by itself 8. Fear of HIV test 9. I don’t know service is provided there 10. I thought the cost is too expensive 11. Mistrust of health services provision 12. My belief system don’t allow 13. Other (specify)-------------------- | |  |  |
| 206 | Which HCF did you first visit? | | 1. Health post 2. Public Hospital 3. Health center 4. Private (Hospital/Clinic) 5. Others (specify-------- | |  |  |
| 207 | How far is the first HCF you visited from your usual residence? | | ____________minute/hour walking distance/transport  ____________Km | |  |  |
| 208 | In total how long time, did you spend from the onset of illness to the first HCF visit? | | __________________days/weeks/months | |  | |
| 209 | When was your final diagnosis of TB made? | | ------------------------------------dd/mm/yyyy | |  |  |
| 210 | Where was the final diagnosis of TB made? | | 1. Health center 3. Private clinic 2. Hospital 4. Other | |  |  |
| 211 | Until the final diagnosis of TB, how many healthcare facilities did you visit? | | ___________________ | |  |  |
| 212 | How many visits did you made to HCF until the final diagnosis of TB? | | ----------------------- | |  |  |
| 213 | In total how long time did you spend from the onset of your illness to diagnoses of TB? | | ___________________days/weeks/months | |  |  |
| 214 | How long time did you spend since you first visited HCF until TB diagnosis? | | ___________________days/weeks/months | |  |  |
| 215 | In your family, does anyone have an illness like you? | | 1. Yes (how many---------- 2. No | | 218 |  |
| 216 | Did they seek care for it? | | 1. Yes 2. No | | 218 |  |
| 217 | Why they did not seek care?  **Note for interviewer: More than one response possible and check for that without reading options** | | 1. Took from my medicine 2. Expensive healthcare fee 3. Refused 4. Other (specify----------------- | |  |  |

| Visit | Name of  Providers/ institution where  treatment or advice sought | Total time spent (in hours, including travel ) | Accompanies (Y/N) (# of accompanies | Consultation /card charge | All test costs  (for sputum, blood, stool, urine or other specify----) | X ray/ US or other diagnostic costs | Total drug costs  (all kinds | Travel  Costs  (round trip including accompany if any | Food  costs  (total | Accommodation costs if any | Any other costs(specify | Total costs  per visit |
| --- | --- | --- | --- | --- | --- | --- | --- | --- | --- | --- | --- | --- |
| Visit 1 |  |  |  |  |  |  |  |  |  |  |  |  |
| Visit 2 |  |  |  |  |  |  |  |  |  |  |  |  |
| Visit 3 |  |  |  |  |  |  |  |  |  |  |  |  |
| Visit 4 |  |  |  |  |  |  |  |  |  |  |  |  |
| Visit 5 |  |  |  |  |  |  |  |  |  |  |  |  |
| Visit 6 |  |  |  |  |  |  |  |  |  |  |  |  |
| Visit 7 |  |  |  |  |  |  |  |  |  |  |  |  |
| Visit 8 |  |  |  |  |  |  |  |  |  |  |  |  |
| Visit 9 |  |  |  |  |  |  |  |  |  |  |  |  |
| Total | | | | | | | | | | | |  |

**Cost of seeking care and diagnosis**

218 About how much did you spend in ETB for each of the visits before you were diagnosed with TB, including the visit when you actually received your diagnosis? *For all that do not apply, mark N/A; Fill one line per visit*

**Note for interviewer**: provider could be traditional healer, holy water priest, any healthcare facility.

Time elapsed is time in minute/hours spent for round trip travel and waiting time at the provider

Drug is any medication prescribed and taken at each visits to provider

| **Part III; Anti-TB treatment practices and patient cost of care** | | | |
| --- | --- | --- | --- |
| Q no | Question | Options /response | Skip |
| 301 | When did you start anti-TB treatment? | _____________________dd/mm/yy |  |
| 302 | How long it takes you to start TB treatment since first HCP consultation. | --------------------days/weeks/months |  |
| 303 | How long after the diagnosis of TB that you commenced anti-TB treatment? | 1. Immediately 2. ---------------------days/weeks | 305 |
| 304 | Why you did not start treatment immediately? | 1. I was reluctant to initiate the treatment 2. Fear of long treatment 3. Lack of anti-TB drugs at the facility 4. TB clinic was closed 5. Absence of DOT provider 6. Failure to present treatment supporter 7. Inability to arrange accommodation at nearby 8. Too ill to initiate early 9. Other (specify--------------------------- |  |
| 305 | At which health facility did you start the anti-TB treatment? | 1. In this facility 2. Other facility(specify------ | 308 |
| 306 | If started elsewhere, why you did you start treatment there? | 1. It was must to start at diagnosed HCF 2. I was too ill and told to be admitted there 3. Treatment was not available here at the time 4. I was near to that facility at the time 5. Other specify--------------- |  |
| 307 | For how long did you take the treatment there? | ______________days/weeks/months |  |
| 308 | How were you taking the anti-TB drugs? | 1. Hospitalized 2. Ambulatory | 312 |
| 309 | For how long did you stay at the hospital? | ______________days/weeks/months |  |
| 310 | Did someone accompany you during hospitalization? | 1. Yes (for -----------------days/weeks/months 2. No |  |
| 311 | During the hospitalization, how much did you pay in total for the following items? | 1. Food ------------ 2. Accommodation --------- 3. Laboratory tests------------ 4. Drugs ------------ 5. Service charge--------- 6. Other(specify------ 7. I did not pay 8. I do not remember 9. Did not pay (its free or covered by specify------- |  |
| 312 | How far is this facility from your residence? | ------------minutes/hour walk/vehicle -------Km |  |
| 313 | How long time did you spend for a single visit including round trip travel and waiting time? | Round trip travel-----minutes/hour walking/vehicle  waiting time at facility____minute/hour total----------- |  |
| 314 | Were your family/ other accompanied you or gone in your place to pick anti-TB drugs? | 1. Yes (in how many visits----------------- 2. No | 316 |

| Part IV: **Indirect costs of care seeking and diagnosis to patients and escorts** | | | | |  |
| --- | --- | --- | --- | --- | --- |
| 401 | What is your formal work? | | 1. Formal government/private employee 2. Self employed 3. Farmer 4. Daily laborer 5. Student 6. Other (specify)-- | | 403 |
| 402 | | How much salary do you earn monthly? | 1. _____________ETB | |  |
| 403 | | On average, how much do you earn monthly from your regular work? | 1. _____________ETB | |  |
| 404 | | Do you regularly perform your works? | 1. Yes 2. No | | 406 |
| 405 | | Why did not you regularly work? | 1. Due to the TB illness 2. Other reasons (specify------------ | | 406 |
| 406 | | When was the last time you were working your regular work? | ----------------dd/mm/yy  ---------------days/weeks/months from now | |  |
| 407 | | On average, how many hours did you work per day **BEFORE** you became ill with TB? |  | |  |
| 408 | | On average, how many hours per day do you work after you became ill with TB? |  | |  |
| 409 | | If work hour is different, is the change related to the TB illness? | 1. Yes 2. No | |  |
| 410 | | Have you ever stopped working/going to school/doing housework due to TB illness? | 1. Yes 2. No | | 413 |
| 411 | | If YES: for how long did you quit working? | --------------------------days/weeks/months | |  |
| 412 | | Who covers the work that you used to work? | 1. Family member 2. Delegate office mate/co-worker 3. Hired person 4. Nobody 5. Other specify------- | |  |
| 413 | | How much ETB did you estimate the loss of income due to lost workdays? | ___________________________________ | |  |
| 414 | | Did someone quit his or her regular work specifically to take care/accompany you? | 1. Yes (how many------------ 2. No | | 501 |
| 415 | | What was the main occupation of your accompanied caregiver? | 1. Government/private employed (monthly salary--- 2. Farmer 3. Daily laborer (daily wage-------- 4. Student 5. Merchant 6. No work 7. Other (specify)-- | |  |
| 416 | | For how long did they quit their work? | ___________days/weeks/months | |  |
| 417 | | How much ETB would you estimate have they lost while taking care of you? | _______________________________ETB | |  |
| ***Part V: Household characteristics, assets, Income and Spending*** | | | | |  |
| 501 | | Who is the head of the household? | | 1. Patient 2. Husband/father 3. Wife/mother 4. Brother/sister 5. Other specify---------- |  |
| 502 | | Who is the primary income earner in the household? *Circle most appropriate* | | 1. Patient 4. Wife/mother 2. Husband/father 5. Extended family 3. Son/daughter 6. Other (specify |  |
| 505 | | How many people regularly sleep in your house? | |  |  |
| 507 | | How much do you estimate was the average annual income of your household (for all persons in the house, including patient) | | 1. Income of patient: ------------- 2. Other household member------------ 3. Other income : --------------   TOTAL: ---------------- |  |
| 508 | | Do you think that your TB illness reduced the total household income? | | 1. Yes 2. No |  |
| 509 | | Besides yourself, does anyone else of your household receive treatment for TB? | | 1. Yes 2. No |  |

| **Part VI; Coping mechanisms for costs due to TB illness** | | |  |
| --- | --- | --- | --- |
| 601 | How much do you estimate that your household incurred your TB care seeking? | 1. --------------------ETB 2. I do not Know |  |
| 602 | How would you describe the expenditure for your TB care? | 1. Expensive 2. Fair 3. Cheap 4. Free |  |
| 603 | How did you cover the costs incurred for TB illness and care seeking?  **Note**: **more than one response is possible and put amount of money generated with each coping mechanisms** | 1. Sold assets/products 2. Received financial assistance from outside---- 3. Withdrew saving---------------- 4. Borrow money--------------- 5. Changed jobs 6. Reduced food consumption 7. Drop out of school 8. Other specify-------------------- | 607 |
| 604 | If you sold assets, what did you sell? *Circle most appropriate* | 1. Land 5. Vehicle 2. Livestock 4. Farm produce 3. Household item 6. Other---- |  |
| 605 | What is the estimated market value of the property you sold? | __________________ETB |  |
| 606 | How much did you earn from the sale? | ______________________ETB |  |
| 607 | Has the TB illness affected your social or private life in any way? | 1. Yes 2. No | 609 |
| 608 | If Yes, what did it brought on you? | 1. Divorce 2. Disruption of sexual life 3. Drop out of school 4. Loss of Job 5. Reduced income 6. Other------ |  |
| 609 | If the government could provide you with some service to ease the burden of TB on you and your household, what would you prefer to have? ***State options, choose one*** | 1. Transport vouchers 2. food vouchers 3. More efficient service 4. Other (specify): |  |

**አዲስ አበባ ዩኒቨርሲቲ**

**ጤና ሳይንስ ኮሌጅ**

**ህ/ሰብ ጤና ት/ቤት**

**የቲቢ በሽታ ህክምና መዘግየት፣ ወጪዉና የህክምና ዉጤት የስድስት ወራት ህክምና ከመጀመሩ በፊትና በ|ላ፤ ለፍልስፍና ዶክቴሬት ዲግሪ በህ/ሰብ ጤና ማJያ ምርምር የጥናት ተሳታፊ ህሙማን መጠይቅ**

**የጥናት ተሳታፊዎች ስምምነት መቀበያ**

ጤና ይስጥልኝ! ---------------እባላለሁ፡፡ከአዲስ አበባ ዩኒቨርሲቲ የጥናት ቡድን ጋር የሚሰራ ሲሆን በሳይንሳዊ ዜዴ የተመረጡ ቲቢ ህሙማንን በበሽታዉ የምርመራና የህክምና ልምዶቻቸዉ እና ወጪያቸዉ ዙሪያ እንጠይቃለን፡፡ የጥናቱም ዓላማ ለቲቢ በሽታ ምርመራና ህክምና መዘግየት ምክንያቶችንና ተያያዠ ወጪዎችን በመለየት የመፍትሄ አቅጣጫዎችን መጠቆም ነዉ፡፡ ስለሆነም ለታሰበዉ ጥናት በጣም ጠቃሚ የሆኑ መረጃዎችን ስለምጠይቅዎት የሚሰጡኝ እዉነተኛ መረጃ ለሚታቀደዉ መፍትሄ ወሳኝ ሚና ይኖረዋል፡፡ በማናቸዉም ጉዳዮች ዙሪያ የሚሰጡኝ መረጃ ምስጥራዊነቱ የተጠበቀ ነዉ፤ ለዚሁም ማንነትዎን የሚገለጽ ማንኛዉም መረጃ የማይያያዝ ከመሆኑም ባሻገር መረጃዉ ከጥናቱ ዓላማ ዉጭ ለሌላ ወገን ወይም ጉዳይ የማይዉል መሁኑን እናረጋግጣለን፡፡ መጠይቁ 30 ደቂቃ የሚፈጅ ሲሆን መመለስ የማይፈልጉት ጥያቄ ካለ አለመመለስ ወይም በፈለጉበት ጊዜ ማቆም ይችላሉ፡፡ስለተሳትፎዎ እጅግ አድርገን እያመሰገንን ስለጥናቱ ማንኛዉንም መረጃ ከፈለጉ በሚከተሉት አድራሻዎች መጠየቅ ይችላሉ፡፡

1. አቶ አብዮት አስረስ ዋና አጥኚ ፡ ስልክ ቁጥር፡ 0911905554
2. ዶ/ር ዋቅጋሪ ዴረሳ የጥናቱ አማካሪ ፡ ስልክ ቁጥር 0911483714
3. ዶ/ር ደጉ ጀረኔ የጥናቱ አማካሪ ፡ ስልክ ቁጥር 0911546407
4. አድስ አበባ ዩኒቨርሲቲ ጤና ሳይንስ ኮሌጅ የጥናት ስነምግባር ገምጋሚ ቦርድ፡ ስልክ ቁጥር 0115512876

ሀ. መረጃዉና የጥናቱ ዓላማ ግልጽ ነዉ ? 1. አዎን 2. አይደለም-----------በድጋሜ አብራራ/ሪ

ለ. በጥናቱ ለመሳተፍ ፈቃደኛ ነዎት?

1. አዎን----------------ፊርማ------------------ጥያቀዉን ቀጥል/ይ
2. አይደለም------------አቁም/ሚና ወደ ቀጣይ ታማሚ ሂድ/ጅ

ሐ. ስምምነቱ በጥናቱ ተሳታፊ ስለመሰጠቱ የጠያቂዉ ማረጋገጫ

የጠያቂዉ ስም------------------------------------------------------------ፊርማ---------------------ቀን-------------------------

| - 1. **የመጠይቁ አጠቃላይ መግለጫ** | | | | | | | |
| --- | --- | --- | --- | --- | --- | --- | --- |
| የመጠይቅ መለያ |  |  |  | |  |  |  |
| ዞን | 1. ካፋ 2. በንች ማጂ 3. ሸካ | | | | | | |
| ወረዳ | 1. ቦንጋ ከተማ 4. ሚዛን አማን 8. ቴፒ ከተማ 2. ደቻ 5. ሰሜን በንች 9. የኪ 3. ጨና 6. ሸይ በንች 10. ማሻ   7. ሜኢኒትጎልድያ | | | | | | |
| የጤና ተkሙ ስም |  | | | | | | |
| የጤና ተkሙ ዓይነት | 1. ሆስፒታል 2. ጤና ጣቢያ 3. ጤና ኬላ 4. ሌላ ይጠቀስ------------ | | | | | | |
| ተጠያቂዉ | 1. ታማሚዉ 2. የታማሚዉ ቤተሰብ 3. የህክምና አጋዥ/ረዳት 4. ሌላ------- | | | | | | |
| መጠይቁ የተሞላበት ቀን |  | | | የቀጣይ ቀጠሮ ቀን፡______________________________________ | | | |
| የጠያቂዉ/መረጃ ሰብሳቢ ስምና ፊርማ |  | | | የተቆጣጣሪዉ ስምና ፊርማ፡ __________________________________ | | | |

| - 1. **Medical record review checklist** | | | | | | | | | | | | | |
| --- | --- | --- | --- | --- | --- | --- | --- | --- | --- | --- | --- | --- | --- |
| Medical record number | _________________ | | | Date of recent visit:________________________ | | | | | | | | | |
|  |  |  |  | Duration of illness (dd,week,mm) | | | | | | | | | |
| Chief complaint | 1. Cough | | |  | | | | | | | | | |
|  | 1. Fever | | |  | | | | | | | | | |
|  | 1. Chest pain | | |  | | | | | | | | | |
|  | 1. Night sweating | | |  | | | | | | | | | |
|  | 1. Swelling over neck | | |  | | | | | | | | | |
|  | 1. Other specify----------------------- | | |  | | | | | | | | | |
| Key physical findings | 1. HEENT | | |  | | | | | | | | | |
|  | 1. LGS | | |  | | | | | | | | | |
|  | 1. Chest | | |  | | | | | | | | | |
|  | 1. CVS | | |  | | | | | | | | | |
|  | 1. Abdomen | | |  | | | | | | | | | |
|  | 1. Other specify------------------------- | | |  | | | | | | | | | |
| Investigations requested ,their results and unit cost | Investigation | | | Result | | | | Unit cost of the investigation | | | | | |
|  | 1. Sputum for AFB | | |  | | | |  | | | | | |
|  | 1. Xpert MTB/RIF | | |  | | | |  | | | | | |
|  | 1. CXR(chest x ray) | | |  | | | |  | | | | | |
|  | 1. ESR | | |  | | | |  | | | | | |
|  | 1. WBC | | |  | | | |  | | | | | |
|  | 1. Other specify | | |  | | | |  | | | | | |
| Diagnosis/Assessement | Diagnosis | | | Date of diagnosis | | | | | | | | | |
|  | 1. TB | | |  | | | | | | | | | |
|  | 1. Pneumonia | | |  | | | | | | | | | |
|  | 1. B.Asthma | | |  | | | | | | | | | |
|  | 1. Other specify------------------------- | | |  | | | | | | | | | |
|  | 1. Not stated/written | | |  | | | | | | | | | |
| Treatments/plan prescribed and their unit cost |  | | | Unit cost of the treatment precribed | | | | | | | | | |
|  |  |  |  |  | | | | | | | | | |
| - 1. **Unit TB register review** | | | | | | | | | | | | | |
| TB unit number | |  | **Wereda** | | |  | | | | | | **kebele** |  |
| Sex | | 1. Male 2. Female | | | | | | | | | | | |
| Age | | ________________years | | | | | | | | | | | |
| Smear result | | 1. Positive 2. Negative 3. Not done | | | | | | | | **Lab number**:__________ | | | |
| Baseline/intial weight (Kg) | | _________________ | | | **Height (cm)** | | ______________ | | | | | | |
| Patient category | | 1. New 2. Transfer in 3. Other 4. Other specify_________________ | | | | | | | | | | | |
| Type of TB | | 1. PPos 2. Pneg 3. EPTB | | | | | | | | | | | |
| Intensive drugs | | 1. RHZE 2. SRHZE 3. Other ----- | | | | | | | Dose (#of tabs):________ | | | | |
| Treatment started date/dd/mm/yy | | _____________________ | | | | | | | | | | | |
| Xpert MTB/RIF result | | 1. Positive 2. Negative 3. Not done | | | | | | | | | Lab serial #: | | |
| HIV test offered | | 1. Yes 2. No | | | | | | | | | | | |
| HIV test performed | | 1. Yes 2. No | | | | | | | | | | | |
| Place HIV test performed | | 1. OPD 2. Lab 3. TB clinic 4. Other ---- | | | | | | | | | | | |
| HIV result | | 1. Reactive 2. Non reactive 3. NA | | | | | | | | | | | |
| CPT started date | | 1. Yes 2. No if yes when-----------------dd/mm/yy | | | | | | | | | | | |
| Date HIV care enrolled | | Yes 2. No if yes when-----------------dd/mm/yy | | | | | | | | | | | |
| Date ART started | | Yes 2. No if yes when-----------------dd/mm/yy | | | | | | | | | | | |
| ART unique number | |  | | | | | | | | | | | |

| ተቁ | ጥያቄ | | ምላሽ/አማራጭ | | | | ወደ |  |
| --- | --- | --- | --- | --- | --- | --- | --- | --- |
| 101 | kሚ የመኖሪያ አድራሻዎ የት ነዉ? | | 1. ከተማ 2. ገጠር (ወረዳ_____________________ ቀበሌ_____________________ | | | |  |  |
| 102 | የትዳር ሁኔታ እንዴት ነዉ? | | 1. ፈፅሞ ያላገባ/ች 3 ባል/ሚስት የሞተበት/ባት 2. ያገባ/ች 4. የተፋታ/ች/የተለያዩ | | | |  |  |
| 103 | ሃይማኖትዎ ምንድን ነዉ? | | 1. ኦርቶዶክስ ክርስቲያን 4. ፕሮቴስታንት 2. ሙስሊም 5. ባህላዊ 3. ካቶሊክ 6. ሌላ------------------- | | | |  |  |
| 104 | የትኛዉ ብሄረሰብ አባል ነዎት? | | 1. ካፍቾ 4. ሸኮ 7. ኦሮሞ 2. ቤንች 5. አማራ 8. ሜኒት 3. ሸካቾ 6. ትግሬ 9. ሌላ----------------- | | | |  |  |
| 105 | የጨረሱት ከፍተኛ የት/ርት ደረጃ ? | | 1. ምንም ያልተማረ 4. ሁለተኛ ደረጃ ያጠናቀቀ(9-12) 2. የመጀመሪያ ሳይክል ያጠናቀቀ/ች (1-4) 5. የተመረቀ /ሰርትፍኬት/ዲፕሎማ/ዲግሪ 3. ሁለተኛ ሳይክል ያጠናቀቀ/ች (5-8) | | | |  |  |
| 106 | ዋና/መደበኛ ስራዎ ምንድነዉ? | | 1. የመንግስት ተቀጣሪ 6. የቤት እመቤት 10. ነጋዴ 2. የግል ተቀጣሪ 7. ተማሪ 11. ሌላ------------------- 3. በግል ስራ የሚተዳደር 8. አርሶ አደር 4. የቤት ሰራተኛ 9. የቀን ሰራተኛ | | | |  |  |
| **ክፍል ሁለት፡ የህመም አጀማመር፣ ህክምና ዕርዳታ አፈላለግ ልምድና ወጪያቸዉ** | | | | | | |  |  |
| 201 | | አሁን እየታከሙ ያሉት የቲቢ ህመም መቼ ነበር የጀመርዎት? | | 1. ________ቀን/ወር/ዓም/ 2. ____በፊት 3. አላስታዉስም | | |  |  |
| 202 | | መጀመሪያ የታየብዎት ዋና ምልክት/ቶች ምን/ምን ምን ነበር/ሩ? መቼ ነበር ምልክቶቹ የታዩት?  ***ከአንድ በላይ ምላሽ ልኖረዉ ይችላል/ ምርጫ አይነበብም ሌላስ በማለት ተጨማሪ ይጠየቅ*** | | የታዩ ምልክቶች | የጀመረበት ጊዜ/ቀን/ወር/ዓም | |  |  |
|  |  |  |  | 1. ሳል |  | |  |  |
|  |  |  |  | 1. ትኩሳት: |  | |  |  |
|  |  |  |  | 1. የምግብ ፍላጎት መቀነስ |  | |  |  |
|  |  |  |  | 1. ደም የቀላቀለ ሳል |  | |  |  |
|  |  |  |  | 1. ክብደት መቀነስ |  | |  |  |
|  |  |  |  | 1. የደረት ህመም/ዉጋት |  | |  |  |
|  |  |  |  | 1. ማታ ማታ ማላብ |  | |  |  |
|  |  |  |  | 1. ሌላ ይጠቀስ---------------- |  | |  |  |
| 203 | | የትኛዉ ምልክት ነበር መፍትሄ/ የህክምና እርዳታ እንዲፈለጉ ያደረግዎት? ***ከአንድ በላይ ምላሽ ልኖረዉ ይችላል፤*** ***የ202 ምላሽ አስታዉስ/ሽ ወይም ከ202 ምላሽ ዉስጥ አስመርጥ/ጪ*** | | 1. ሳል 5. ትኩሳት 2. ክብደት መቀነስ 6. የደረት ህመም. 3. ደም የቀላቀለ ሳል 7. ማታ ማታ ማላብ 4. የምግብ ፍላጎት መቀነስ 8. ሌላ----------- | | |  |  |
| 204 | | ህመሙ እንደጀመርዎት **መጀመሪያ** ምንድነዉ ያደረጉት ወይም ምን እርምጃ ነዉ የወሰዱት? መፍትሄዉን ወይም እርምጃዉን መቼ ነበር የወሰዱት?  ***ምርጫ አይነበብም፤ አንድ ምላሽ ብቻ ይኖረዋል፡፡*** | | **የተወሰደ መፍተሄ** | | **የተወሰደበት ጊዜ/ቀን/ወር/ዓም** | 206 | |
|  |  |  |  | 1. በራሴ መድሃኒት ገዝቼ ዋጥኩ | |  |  |  |
|  |  |  |  | 1. የባህል ህክምና ቦታ ሄድኩ | |  |  |  |
|  |  |  |  | 1. ጸበል ቦታ ሄድኩ/ጠጣሁ | |  |  |  |
|  |  |  |  | 1. የጤና ኤክሰቴንሽን አማከርኩ | |  |  |  |
|  |  |  |  | 1. ጤና ተkም /የግል ክልኒክ፣ የመንግስት ጤና ከላ፣ጤና ጣቢያ፣ ሆስፒታል ሄድኩ/ | |  |  |  |
|  |  |  |  | 1. ሌላ-------------------------- | |  |  |  |
| 205 | | ህመሙ እንደጀመርዎት መጀመሪያ ወደ ጤና ተkም ያልሄዱት ለምንድን ነዉ?  ***ጤና ተkም ማለት የግል ክልኒክ ወይም ጤና ኬላ ወይም ጤና ጣቢያ ወይም ሆስፒታልን ያካትታል፡፡***  ***ከአንድ በላይ ምላሽ ሊኖረዉ ይችላል፤ምርጫ አይነበብም፣ ሌላስ በማለት ተጨማሪ ይጠየቅ*** | | 1. ህመሙ በራሱ ይተዋል ብዬ 2. ጤና ተkማቱ ስለራቁኝ 3. ጤና ተkማቱ አገልግሎት ስለማይሰጡ 4. በተkማቱ አገልግሎቱን ለማግኘት ረጅም ጊዜ ስለሚፈጅ 5. በተkማቱ መጥፎ ገጠመኝ ስለነበረኝ 6. የምርመራ ዉጤቱ ቲቢ እንዳይሆን ፈርቼ 7. ቲቢ የተለመደና በራሱ የሚድን በሽታ ስለሆነ 8. የኤች አይ ቪ ምርመራ ፈርቼ 9. የህክምና ወጪ ዉድ ስለሆነ 10. የጤና ባለሙያዎችን ስለማላምንባቸዉ 11. ሌላ ይጠቀስ-------------------- | | |  |  |
| 206 | | የትኛዉ ጤና ተkም ነበር መጀመሪያ የሄዱት?  ***ጤና ተkም የግል ክሊኒክ ወይም ጤና ኬላ ወይም ጤና ጣቢያ ወይም ሆስፒታልን ያካትታል፣፣*** | | 1. ጤና ኬላ 4. የግል ሆስፒታል/ክሊኒክ 2. መንግስት ሆስፒታል 5. ሌላ -------- 3. ጤና ጣቢያ | | |  |  |
| 207 | | ይህ የሄዱበት ጤና ተkም ከቤትዎ ምን ያህል ይርቃል? | | _______ሰዓት የእግር መንገድ/ኪሜ/ | | |  |  |
| 208 | | ወደ ጤና ተkሙ ማለትም /የግል ክሊኒክ፣ የመንግስት ጤና ኬላ፣ጤና ጣቢያ፣ ሆስፒታል/ ለመሄድ እንዴት ተነሳሱ ወይም ምን ገፋፍትዎት ነዉ ሊሄዱ የቻሉት?  ***ከአንድ በላይ ምላሽ ልኖረዉ ይችላል፤ምርጫ አይነበብም፣ ሌላስ በማለት ተጨማሪ ይጠየቅ*** | | 1. ከመድሃኒት ቤት/መደብር ተልኬ 7. በራሴ ተነሳስቼ 2. የጤና ኤክስቴንሽን ባለሙያ ልካኝ 8. በባህል ሃኪም ተመክሬ 3. ከኤች አይ ቪ ክፍል ተልኬ/ሪፈር ተደርጌ 4. ከጸበል ቦታ ተልኬ/ተመክሬ 5. በህክምና ላይ ባለ/ች ቲቢ ታማሚ ተመክሬ 9. ሌላ---------- 6. ዘመዶቼ/ቤተሰቦቼ መክረዉኝ | | |  |  |
| 209 | | ለህመሙ በመጀመሪያ ጊዜ ወደ ጤና ተkሙ የሄዱት መቼ ነዉ? | | _______________________________________________ቀን/ወር/ዓም | | |  |  |
| 210 | | ህመሙ ከጀምርዎት ከምን ያህል ጊዜ በ\|ላ ነዉ መጀመሪያ ወደ ጤና ተkም የሄዱት? | | -------------------------------ቀናት/ሳምንት/ወራት | | |  | |
| 211 | | ህመሙ እንደጀመርዎ ወደ ጤና ተkም ለመሄድ የዘገዩ ይመስልዎታል? | | 1. አዎን 2. አይደለም | | | 213 |  |
| 212 | | የዘገዩ ከሆነ ወደ ጤና ተkም ለመሄድ የዘገዩበት ምክንያትዎ ምንድነዉ?  ***ምርጫ አይነበብም፣ ሌላስ በማለት ተጨማሪ ይጠየቅ*** | | 1. ህመሙ በራሱ ይተዋል ብዬ ተስፋ በማድረግ 2. ከማህበረሰቡ መገለል እንዳይደርስብኝ ፈርቼ 3. በሽታዉ ቲቢ እንዳይሆን ፈርቼ 4. በጤና ተkም የሚደረግ ኤች አይ ቪ ምርመራ ፈርቼ 5. የገንዘብ ችግር ስለገጠመኝ 6. በጤና ተkም ያሉ ባለሙያዎች አመለካከት ጥሩ ስላልሆነ 7. በጤና ተkም የሚሰጡ አገልግሎት ጥራት ስለሌላቸዉ 8. ሌላ ይጠቀስ----------------------------------- | | |  |  |
| 213 | | **መጀመሪያ የሄዱበትን** ጤና ተkም ለምን መረጡ?    **የ*206ን ምላሽ አስታዉስ/ሽ ከአንድ በላይ ምላሽ ሊኖረዉ ይችላል/ ምርጫ አይነበብም፣ ሌላስ በማለት ተጨማሪ ይጠየቅ*** | | 1. ቅርብ ስለሆነ 2. አገልግሎቱ ሁል ጊዜ/በማንኛዉም ጊዜ ሰለሚገኝ 3. አገልግሎቱ በነጻ ስለሚሰጥ 4. በተkሙ እንደምድን ስለተማመንኩ 5. በሌላ ሰዉ ተመክሬ 6. በተkሙ ሚስጥር ስለሚጠበቅ 7. ብቁ ባለሙያዎች በተkሙ ስለሚገኙ 8. የተJላ የምርመራ መሳሪያዎች በተkሙ ስለሚገኙ 9. ሌላ ይጠቀስ----------------------- | | |  |  |
| 214 | | መጀመሪያ ጤና ተkም በሄዱበት ወቅት የህመምዎ የምርመራ ዉጤት ምን ነበር? | | 1. ጉንፋን 4. ቲቢ 2. ሳንባ ምች 5. አልተነገረኝም/አላዉቅም 3. አስም 6. ሌላ------------------------ | | | 218 |  |
| 215 | | ወደ ሌላ ተkም ሪፈር ተደርገዉ ወይም በራስዎ ሄደዉ ነበር? | | 1. አዎን 2. አይደለም | | | 218 |  |
| 216 | | ወደ የትኛዉ ተkም ነበር የተላኩትወይም በራስዎ የሄዱት? | | 1. ጤና ጣቢያ 2. ሆሰፒታል 3. ሌላ ይጠቀስ------ | | |  |  |
| 217 | | ለምን ነበር ሪፈር የተደረጉት ወይም በራስዎ የሄዱት? | | 1. ላቦራቶሪ ስላልነበር 4. በሽታዬን ማወቅ ስላልቻሉ 2. ኤክስሬይ ስላልነበር 5. ሌላ ይጠቀስ-------------- 3. ተkሙ የቲቢ ምርምራና ህክምና ስለማይሰጥ | | |  |  |
| 218 | | ህመምዎ ቲቢ መሆኑ የተረጋገጠዉ መቼ ነበር? | | _____________ቀን/ወር/ዓም / ------------ቀናት/ሳምንት/ወር በፊት | | |  |  |
| 219 | | ህመምዎ ቲቢ አንደሚሆን ጠርጥረዉ ነበር? | | 1. አዎን 2. አይደለም | | |  |  |
| 220 | | ህመምዎ ቲቢ ነዉ ሲባሉ ምን ተሰማዎ? | | 1. ፍርሃት 4. ተስፋ መቁረጥ 2. ሃፍረት 5. ሌላ----------------- 3. ምንም አልተሰማኝም | | |  |  |
| 221 | | ህመምዎ ቲቢ መሆኑ የተረጋገጠዉ የት ነበር? | | 1. ጤና ጣቢያ 3. የግል ክሊኒክ/ሆስፒታል 2. ሆስፒታል 4. ሌላ------- | | |  |  |
| 222 | | ቲቢ እስኪረጋገጥ ድረስ በአጠቃላይ **ወደ ስንት ጤና ተkማት** ሄዱ? | | ___________________ ***የሄዱባቸዉን ተkማት ብዛት ያስታዉሱ*** | | |  |  |
| 223 | | ቲቢ እስኪረጋገጥ ድረስ በአጠቃላይ ወደ ጤና ተkም **ስንት ጊዜ** ተመላለሱ? **/የሄዱባቸዉን ተkማት በማስታወስ እርዳ/ጂ** | | ----------------------- ***በየተkማቱ ያደረጉትን ምልልስ ብዛት ይጠይቁ*** | | |  |  |
| 224 | | በአጠቃላይ **ህመሙ ከጀመርዎ አንስቶ ቲቢ እስኪረጋግጥ** ድረስ ምን ያህል ጊዜ ፈጀብዎ? | | ___________________ቀናት/ሳምንት/ወራት | | |  |  |
| 225 | | ለህመሙ **የጤና ተkም መጀመሪያ ከሄዱ ጊዜ አንስቶ ቲቢ እስኪረጋገጥ** ምን ያህል ጊዜ ፈጀብዎ? | | ___________________ቀናት/ሳምንት/ወራት | | |  |  |
| 226 | | ወደ **ጤና ተkም ከሄዱ በ\|ላ ቲቢ እስኪረጋገጥ**የዘገዩ ይመስሎታል? | | 1. አዎን 2. አይደለም | | | 228 |  |
| 227 | | የዘገዩ ወይም ረጅም ጊዜ የወሰደ ከመሰልዎት የዘገየበት ወይም ቶሎ ያልተረጋገጠበት ምክንያት ምንድነዉ ይላሉ?  ***ምርጫ አይነበብም፣ ሌላስ በማለት ተጨማሪ ይጠየቅ*** | | 1. ባለሙያዎች በሽታዉን መለየት ስላልቻሉ 2. ለምርመራ የሚያስፈልጉ ግብዐቶች እጥረት 3. አላስፈላጊ መድሃኒቶች መስጠታቸዉ 4. ወደ ተለያዩ ጤና ተkማት ሪፈር ሲደረግ ቆይቼ 5. ሌላ ይጠቀስ------------------------------------ | | |  |  |
| 228 | | በቤተሰብዎ ዉስጥ የእርስዎን ዓይነት ህመም የሚያመዉ ሰዉ አለ? | | 1. አዎን/ስንት ናቸዉ----------- 2. አይደለም | | | 231 |  |
| 229 | | የታመመ ካለ ወደ ህክምና ተkም ሄደዋል/ተመርምረዋል? | | 1. አዎን 2. አይደለም | | | 231 |  |
| 230 | | ወደህክምና ያልሄዱት ለምንድነዉ?  ***ምርጫ አይነበብም፣ ሌላስ በማለት ተጨማሪ ይጠየቅ*** | | 1. ከእኔ መድሃኒት ስለወሰዱ 2. የህክምና ወጪዉ ዉድ ስለሆነ 3. ታማሚዎቹ እምቢ ብለዉ 4. ሌላ--------------- | | |  |  |

| ምልልስ | ምርመራ/ህክምና የተሰጠበት ቦታ | የፈጀብዎት ጊዜ(ቆይታ ጉዞን ጨምሮ) | አብሮዎት ሌላ ሰው ነበር(ስንት ሰዎች) | ለካርድ የከፈሉት ብር | ለተለያዩ ላቦራቶሪ ምርመራዎች (ደም፣አክታ፣ሽንት)  }ÅUa ÃVL | ለራጅ ምርመራ | ለመድሃኒት | ለደርሶ መልስ ትራንስፖርት አብሮዎት ከነበሩት ሰዉ ጭምር | ለምግብና መጠጥ መንገድ ላይና ዉጤት ሲጠብቁ | ለአልጋ | ለሌሎች ወጪዎች | አጠቃላይ የአንድ ምልልስ ወጪ |
| --- | --- | --- | --- | --- | --- | --- | --- | --- | --- | --- | --- | --- |
| 1 |  |  |  |  |  |  |  |  |  |  |  |  |
| 2 |  |  |  |  |  |  |  |  |  |  |  |  |
| 3 |  |  |  |  |  |  |  |  |  |  |  |  |
| 4 |  |  |  |  |  |  |  |  |  |  |  |  |
| 5 |  |  |  |  |  |  |  |  |  |  |  |  |
| 6 |  |  |  |  |  |  |  |  |  |  |  |  |
| 7 |  |  |  |  |  |  |  |  |  |  |  |  |
| 8 |  |  |  |  |  |  |  |  |  |  |  |  |
| 10 |  |  |  |  |  |  |  |  |  |  |  |  |
| አጠቃላይ ወጪዎች ድምር በብር | | | | | | | | | | | |  |

የቲቢ በሽታ እስኪረጋገጠልዎ ድረወደ ተለያዩ ቦታዎች ወይም ጤና ተkም በተመላለሱበት ወቅት ቲቢ የተረጋገጠበትን ዕለት ጨምሮ ምን ያህል ከፈሉ፡፡

| **Patient follow up questionnaire** | | | | | | | | | | | | | | | | |
| --- | --- | --- | --- | --- | --- | --- | --- | --- | --- | --- | --- | --- | --- | --- | --- | --- |
| No | Questionnaire ID | |  | | |  | | | |  | |  | |  | |  |
| 001 | Zone | | 1. Kaffa | | | | | Bench-Maji | | | | | Sheka | | |  |
| 002 | Woreda | | 1. Bonga town 2. Decha 3. Chena | | | | | 1. Mizan Aman 2. North Bench 3. Shey Bench 4. Meint Goldiya | | | | | 1. Tepi town 2. Yeki 3. Masha | | |  |
| 003 | Name of health facility | | 1. Hospital 2. Health center 3. Health post 4. Other( specify-------- | | | | | | | | | | | | |  |
| 004 | Date of interview (dd/mm/yy) | |  | | | | | | | | | | | | |  |
| 005 | Name and signature of data collector | |  | | | | | | | | | | | | |  |
| 006 | Name and signature of supervisor | |  | | | | | | | | | | | | |  |
| **Unit TB register review (fill what has been documented after the treatment intiation)** | | | | | | | | | | | | | | | |  |
| 007 | TB unit number |  | | Date anti-TB started__________________dd/mm/yy | | | | | | | | | | | |  |
| 014 | Recorded number of attendance | Intensive phase | | | | | Continuation phase | | | | | | | | |  |
|  |  |  | | | | |  | | | | | | | | |  |
| 015 | Treatment outcome | 1. Cured 5. Treatment Failure 2. Completed 6. Died 3. Lost to follow-up 7. Other specify---- 4. Transferred out | | | | | | | | | | | | | |  |
| 016 | Date outcome ascertained (dd/mm/yy) | | | | | | | |  | | | | | | |  |
| \| **Medical record number (MRN)** \|  \| \| \| \| --- \| --- \| --- \| --- \| \| Number of Visits after TB DX \|  \| \| \| \| Date of Visit (dd/mm/yy) \| Reason for visit /diagnosis \| Investigations/  Treatment prescribed \| Unit cost \| \|  \|  \|  \|  \| \|  \|  \| \|  \|  \| \|  \|  \|  \|  \| \|  \|  \| \|  \|  \| \|  \|  \|  \|  \| \|  \|  \| \|  \|  \|   **Patient follow-up interview questionnaire** | | | | | | | | | | | | | | | | |
| 801 | Date of follow up (dd/mm/yy) | | | |  | | | | | | | | | |  | |
| 802 | Place follow up made | | | | 1. Home 2. Health facility | | | | | | | | | |  | |
| 803 | Outcome of follow up | | | | 1. Patient traced and interviewed 2. Patient transferred to other treatment center 3. Patient lost to follow-up 4. Treatment failed and regimen changed 5. Patient died | | | | | | | | | | Stop | |
| 804 | Person interviewed | | | | 1. Patient 2. Parents/care giver 3. Treatment supporter 4. Other (specify----------------------- | | | | | | | | | |  | |
| 805 | From where were you getting the required drugs? | | | | During intensive phase | | | | | | Continuation phase | | | |  | |
|  |  |  |  |  | 1. This center 2. Health post 3. Other (-------- | | | | | | 1. This center 2. Health post 3. Other (-------- | | | |  | |
| 806 | How frequent were you picking the anti-TB drugs | | | | During intensive phase | | | | | | Continuation phase | | | |  | |
|  |  |  |  |  | 1. Daily 2. Every three days 3. Weekly 4. Other -------------- | | | | | | 1. Daily 2. Every three days 3. Weekly 4. Monthly | | | |  |  |
| 810 | While you visit HCF for collecting anti-TB drugs, how long time you elapse for a single visit | | | | During intensive phase | | | | | | Continuation phase | | | |  | |
|  |  |  |  |  | Round trip travel-------min  Waiting time--------min | | | | | | Round trip travel-------min  Waiting time--------min | | | |  | |
| 811 | During your visits for anti-TB drug collection, how much ETB did pay in total for the following?  **Note: transport, food , accommodation includes those incurred for accompanies if any** | | | | During intensive phase | | | | | | Continuation phase | | | |  | |
|  |  |  |  |  | 1. Service fee---- 2. TB drug-------- 3. Sputum test------ 4. Other lab tests--- 5. Other drugs------ 6. Lodging-------- 7. Food---------- 8. Transport (round trip)- 9. Other ------ | | | | | | 1. Service fee---- 2. TB drug-------- 3. Sputum test------ 4. Other lab tests--- 5. Other drugs------ 6. Lodging-------- 7. Food---------- 8. Transport (round trip)- 9. Other ------ | | | |  | |
| 812 | After you initiated anti-TB treatment, have encountered any other illness? | | | | 1. Yes 2. No | | | | | | | | | | 815 | |
| 813 | Have you sought care for the illness? | | | | 1. Yes 2. No | | | | | | | | | |  | |
| 814 | How much did you pay for the investigations and drugs during the care seeking and treatment? | | | | Consultation------- transport------lodging-----  Laboratory tests------- food ------ other-------  Drugs ----- Total------------------------------- | | | | | | | | | |  | |
| 815 | Have you commenced your regular work? | | | | 1. Yes 2. No | | | | | | | | | | Finish | |
| 816 | When did you start the work? | | | | 1. __________________dd/mm/yy 2. After --------------------days/weeks/months treatment | | | | | | | | | |  | |

| **በቲቢ ህክምና ላይ ያሉ ታማሚዎች መከታተያ መጠይቅ** | | | | | | | | | | | | | | | |
| --- | --- | --- | --- | --- | --- | --- | --- | --- | --- | --- | --- | --- | --- | --- | --- |
| **ተቁ** | የመጠይቅ መለያ | |  | | |  | | | |  | |  |  | |  |
| 001 | ዞን | | 1. ካፋ 2. በንች ማጂ 3. ሸካ | | | | | | | | | | | |  |
| 002 | ወረዳ | | 1. ቦንጋ ከተማ 4. ሚዛን አማን 8. ቴፒ ከተማ 2. ደቻ 5. ሰሜን በንች 9. የኪ 3. ጨና 6. ሸይ በንች 10. ማሻ   7. ሜኢኒት ጎልድያ | | | | | | | | | | | |  |
| 003 | የጤና ተቋሙ ስም | |  | | | | | | | | | | | |  |
| 004 | የጤና ተቋሙ ዓይነት | | 1. ሆስፒታል 2. ጤና ጣቢያ 3. ጤና ኬላ 4. ሌላ ይጠቀስ---------- | | | | | | | | | | | |  |
| 005 | መጠይቁ የተሞላበት ቀን | |  | | | | | | የተቆጣጣሪዉ ስም____________________________ | | | | | |  |
| 006 | መረጃ ሰብሳቢ ስምና ፊርማ | |  | | | | | | ፊርማ፡ ____________________________ | | | | | |  |
| **Unit TB register review (fill what has been documented after the treatment initiation)** | | | | | | | | | | | | | | |  |
| 007 | TB unit number |  | | Date anti-TB started__________________dd/mm/yy | | | | | | | | | | |  |
| 014 | Recorded number of attendance | Intensive phase | | | | | Continuation phase | | | | | | | |  |
|  |  |  | | | | |  | | | | | | | |  |
| 015 | Treatment outcome | 1. Cured 5. Treatment Failure 2. Completed 6. Died 3. Lost to follow-up 7. Other specify---- 4. Transferred out | | | | | | | | | | | | |  |
| 016 | Date outcome ascertained (dd/mm/yy) | | | | | | |  | | | | | | |  |
| \| Medical record number (MRN) \|  \| \| \| \| --- \| --- \| --- \| --- \| \| Number of Visits after TB DX \|  \| \| \| \| Date of Visit (dd/mm/yy) \| Reason for visit /diagnosis \| Investigations/  Treatment prescribed \| Unit cost \| \|  \|  \|  \|  \| \|  \|  \| \|  \|  \| \|  \|  \|  \|  \| \|  \|  \| \|  \|  \| \|  \|  \|  \|  \| \|  \|  \| \|  \|  \|   **የታማሚ ወይም አስታማሚዎች ክትትል መጠይቅ** | | | | | | | | | | | | | | | |
| 801 | ክትትሉ የተደረገበት ጊዜ/ቀን/ወር/ዓም/ | | | |  | | | | | | | | |  | |
| 802 | ክትትሉ የተደረገበት ቦታ | | | | 1. የታማሚ ቤት 2. በጤና ተቀም 3. ሌላ ካለ ይገለፅ---- | | | | | | | | |  | |
| 803 | የክትትሉ ዉጤት | | | | 1. ታማሚዉ ተግኝቶ ተጠይቋል 2. ታማሚዉ ወደ ሌላ ህክምና ጣቢያ ተዛዉሯል /ይጠቀስ----------- 3. ታማሚዉ ህክምናዉን አቋርጧል 4. ታማሚዉ ህክምናዉን ቀይሯል 5. ታማሚዉ ሞቷል | | | | | | | | | አቁም | |
| 804 | ቃለ መጠየቁ የተደረገለት ሰዉ | | | | 1. ታማሚዉ 3. የህክምና ረዳት 2. የቤተሰብ አባል/ይጠቀስ---------- 4. ሌላ ይገለፅ----------------- | | | | | | | | |  | |
| 805 | የሚያስፈልጎትን መድሃኒት መጠን ከየት ነበር የሚያገኙት ? | | | | በመጀመሪያ ሁለት ወራት | | | | | | ቀጣይ አራት ወራት | | |  | |
|  |  |  |  |  | 1. እዝሁ ጣቢያ 2. ጤና ከላ 3. ሌላ -------------------- | | | | | | 1. እዝሁ ጣቢያ 2. ጤና ከላ 3. ሌላ -------------------- | | |  | |
| 806 | የሚያስፈልጎትን መድሃኒት መጠን በምን ያህል ጊዜ ነበር የሚያገኙት ? | | | | በመጀመሪያ ሁለት ወራት | | | | | | ቀጣይ አራት ወራት | | |  | |
|  |  |  |  |  | 1. በየቀኑ 2. በየሶስት ቀን 3. በየሳምንቱ 4. ሌላ ይጠቀስ………. | | | | | | 1. በየቀኑ 2. በየሶስት ቀን 3. በየሳምንቱ 4. በየወሩ | | |  |  |
| 810 | መድሃኒት ለመዉሰድ ሲመላለሱ በአንድ ምልልስ ለጉዞ በመንገድ ላይና መድሃኒት ለመዉሰድ በአጠቃለይ ምን ያህል ጊዜ ይወስድበዎት ነበር? | | | | በመጀመሪያ ሁለት ወራት | | | | | | ቀጣይ አራት ወራት | | |  | |
|  |  |  |  |  | ደርሶ መልስ ጉዞ----------ደቂቃ  በጣቢያዉ-----------------ደቂቃ | | | | | | ደርሶ መልስ ጉዞ----------ደቂቃ  በጣቢያዉ-----------------ደቂቃ | | |  | |
| 811 | **ቲቢ መድሃኒት መዉሰድ ከጀመሩ በኋላ መድሃኒት ለመዉሰድ** ሲመላለሱ እስካሁን ለሚከተሉት ጉዳዮች ምን ያህል ብር ከፈሉ?  **/ትራንሰፖርት፣ ምግብ፣ መኝታ ሌላ ተጨማሪ ሰዉ አብረዉ ከነበሩ የእነሱን ጭምር ይሞላ** | | | | በመጀመሪያ ሁለት ወራት | | | | | | ቀጣይ አራት ወራት | | |  | |
|  |  |  |  |  | 1. አገልግሎት ክፍያ---------- 2. ለቲቢ መድሃኒት--------- 3. ለአክታ ምርመራ--------- 4. ሌላ ላብራቶሪ ምርመራ------ 5. ከቲቢ ዉጭ መድሃኒት ----- 6. ለመኝታ/ማደሪያ------------- 7. ምግብ በመንገድ ላይ-------- 8. ለትራንስፖርት   **/አንድ ደርሶ መልስ** /----------   1. ሌላ------------------ | | | | | | 1. አገልግሎት ክፍያ---------- 2. ለቲቢ መድሃኒት--------- 3. ለአክታ ምርመራ--------- 4. ሌላ ላብራቶሪ ምርመራ------ 5. ከቲቢ ዉጭ መድሃኒት ----- 6. ለመኝታ/ማደሪያ------------- 7. ምግብ በመንገድ ላይ-------- 8. ለትራንስፖርት   **/አንድ ደርሶ መልስ** /----------   1. ሌላ------------------ | | |  | |
| 812 | የቲቢ መድሃኒት መዉሰድ ከጀመሩ በኋላ ሌላ ህመም አመዎት ያዉቃል? | | | | 1. አዎን 2. አይደለም | | | | | | | | | 815 | |
| 813 | ለህመሙ ህክምና እርዳታ አግኝተዉ ነበር? | | | | 1. አዎን 2. አይደለም | | | | | | | | |  | |
| 814 | ለህመሙ ህክምና፣ ምርመራና መድሃኒት ምን ያህል ብር ከፈሉ? | | | | ለካርድ-------- ለትራንስፖርት--------- ሌሎች ወጪዎች---------  ላብራቶሪ-------- ለምግብና መጠጥ------- ድምር---------------  መድሃኒት-------- ለማደሪያ----------- | | | | | | | | |  | |
| 815 | የቲቢ መድሃኒት ከጀመሩ በኋላ የዘወትር ተግባርዎን/ስራዎን ይሰራሉ? | | | | 1. አዎን 2. አይደለም | | | | | | | | | ጨርስ | |
| 816 | መቼ ነዉ የዘወትር ተግባርዎን/ስራዎን የጀመሩት? | | | | ------------------ቀን/ወር----------------ቀናት/ሳምንታት/ ወራት በኋላ | | | | | | | | |  | |
