# Supplementary material for: Pre- and post-diagnosis costs of tuberculosis to patients on Directly Observed Treatment Short course in districts of southwestern Ethiopia: a longitudinal study
Source: J Health Popul Nutr. 2018 May 21;37:15. doi: 10.1186/s41043-018-0146-0 (PMC5963051; doi:10.1186/s41043-018-0146-0)
Supplement: Supplementary file 2 — Table S1. Mean differences of pre, post, and total costs to patients among TB cases on treatment in districts of southwestern Ethiopia January to December 2015. Table S2. Predictors of total cost to patients among TB cases on treatment in districts of southwestern Ethiopia January to December 2015. (DOCX 25 kb) [file 41043_2018_146_MOESM2_ESM.docx]

| Table S1: Mean differences of pre, post and total costs to patients among TB cases on treatment in districts of south west Ethiopia January to December 2015 | | | | | | | |
| --- | --- | --- | --- | --- | --- | --- | --- |
| Variable | | Total pre diagnosis cost(USD) | | Total post diagnosis cost(USD) | | Total cost(USD) | |
|  |  | Mean(SD) | P value | Mean(SD) | P value | Mean(SD) | P value |
| Gender | Male | 112.62(0.12) | 0.1 | 119.73(0.1) | 0.3 | 254.61(0.1) | 0.1 |
|  | Female | 101.25(0.11) |  | 112.51(0.1) |  | 229.12(0.09) |  |
| HIV result | Reactive | 131.2(0.13) | 0.7 | 123.52(0.1) | 0.5 | 287.2(0.11) | 0.1 |
|  | Non reactive | 106.02(0.11) |  | 116.2(0.1) |  | 240.22(0.1) |  |
| Mode of diagnosis | Bacteriological | 97.0(0.11) | 0.001 | 118.25(0.1) | 0.6 | 234.25(0.1) | 0.3 |
|  | Clinical | 121.08(0.12) |  | 115.82(0.1) |  | 255.16(0.1) |  |
| Type of TB | Pulmonary | 97.62(0.14) | F=10.03 | 118.78(0.1) | F=0.52 | 236.16(0.1) | F=3.68 |
|  | Pulmonary negative | 106.9(0.1) | P<0.001 | 111.85(0.1) | P=0.5 | 230.25(0.1) | P=0.03 |
|  | Extra pulmonary | 142.75(0.13) |  | 120.20(0.1) |  | 290.11(0.10) |  |
| Treatment center | Hospital | 121.77(0.13) | 0.005 | 103.05(0.1) | <0.001 | 239.3(0.1) | 0.5 |
|  | Health center | 100.86(0.11) |  | 131.43(0.1) |  | 249.8(0.1) |  |
| Residence | Urban | 114.61(0.12) | 0.08 | 94.02(0.1) | <0.001 | 216.83(0.1) | <0.001 |
|  | Rural | 102.13(0.12) |  | 154.7(0.08) |  | 282.4(0.1) |  |
|  | >22days | 167.11(0.11) |  | 125.28(0.1) |  | 317.39(0.1) |  |
| Action before HCF visit | None | 106.72(0.12) | 0.5 | 111.85(0.11) | 0.006 | 235.89(0.1) | 0.01 |
|  | Took action* | 113.41(0.11) |  | 137.34(0.11) |  | 282.12(0.08) |  |
| First visited HCF | DOTS center | 99.97(0.12) | 0.002 | 118.2(0.1) | 0.6 | 241.61(0.1) | 0.6 |
|  | Non DOTS center | 122.57(0.11) |  | 114.87(0.1) |  | 250.25(0.1) |  |
| Travel time to HCF | <=1hr | 105.8(0.12) | 0.5 | 105.70(0.08) | <0.001 | 227.27(0.09) | <0.001 |
|  | >1hr | 111.20(0.11) |  | 145.14(0.09) |  | 285.25(0.09) |  |
| TB diagnosed HCF | Public | 101.78(0.11) | <0.001 | 116.37(0.1) | 0.6 | 237.9(0.1) | 0.07 |
|  | Private | 153.88(0.13) |  | 121.27(0.11) |  | 292.16(0.1) |  |
| Household annual income category | <=466.93US$ | 110.32(0.11) | 0.6 | 105.24(0.1) | 0.4 | 230.38(0.1) | 0.7 |
|  | >466.93US$ | 118.64(0.12) |  | 128.31(0.1) |  | 276.45(0.1) |  |
| Other co morbidity** | No | 103.92(0.11) | 0.1 | 117.01(0.1) | 0.5 | 238.92(0.1) | 0.3 |
|  | Yes | 135.18(0.13) |  | 116.82(0.1) |  | 274.82(0.11) |  |
| Hospitalized for treatment | Yes | 186.96(0.17) | 0.01 | 240.8(0.09) | <0.001 | 477.85(0.12) | <0.001 |
|  | No | 106.49(0.12) |  | 113.3(0.11) |  | 237.81(0.1) |  |

*=self treatment, consult traditional healer, used holy water, **=HIV, diabetes mellitus,

Hypertension, any forms of cancer, Asthma

Table S2: Predictors of total cost to patients among TB cases on treatment in districts of southwest Ethiopia January to December 2015

| Variable | | | Mean(SD) | Unadjusted exp^a^ coefficient | P value | Adjusted exp coefficient(AeC) | P value |
| --- | --- | --- | --- | --- | --- | --- | --- |
|  |  |  |  | (95% CI) |  | (95% CI) |  |
| Gender | Male | | 254.6(0.1) | Ref. |  | Ref. |  |
|  | Female | | 229.12(0.1) | 0.90(0.79,1.03) | 0.1 | 0.92(0.83,1.02) | 0.1 |
| Educational status | Illiterate | | 248.46(0.08) |  |  |  |  |
|  | Primary | | 250.70(0.09) | 1.01(0.86,1.18) | 0.9 | 0.91(0.78,1.02) | 0.1 |
|  | Secondary and above | | 226.32(0.1) | 0.91(0.75,1.10) | 0.3 | 0.91(0.78,1.05) | 0.2 |
| HIV result | Positive | | 287.20(0.11) | 1.19(0.97,1.48) | 0.1 | 1.15(0.98,1.34) | 0.08 |
|  | Negative | | 240.22(0.1) | Ref. |  |  |  |
| Mode of diagnosis | Bacteriological | | 234.25(0.1) | Ref. |  | Ref. |  |
|  | Clinical | | 255.16(0.11) | 1.09(0.96,1.24) | 0.2 | 1.01(0.92,1.10) | 0.8 |
| Treatment center | Hospital | | 239.30(0.1) |  |  | 0.92(0.83,1.02) | 0.1 |
|  | Health center | | 249.8(0.1) | Ref. |  | Ref. |  |
| Residence | Urban | | 216.83(0.1) | Ref. |  | Ref. |  |
|  | Rural | | 282.40(0.1) | 1.30(1.15,1.48) | <0.001 | 1.24(1.13,1.38) | <0.001* |
| Travel time to treatment center | >1hour | | 227.27(0.09) | 1.26(1.1,1.44) | 0.001 | 1.12(1.01,1.25) | 0.03* |
|  | <=1hour | | 285.25(0.09) |  |  |  |  |
| Patient delay^b^ | | | | 1.005(1.003,1.005) | <0.001 | 1.003(1.002,1.004) | <0.001* |
| Provider delay^b^ | | | | 1.004(1.003,1.004) | <0.001 | 1.003(1.002,1.004) | <0.001* |
| Action before HCF visit | None | 235.89(0.1) | | Ref. |  | Ref. |  |
|  | Took action^c^ | 282.18(0.08) | | 1.19(1.02,1.40) | 0.03 | 1.17(1.04,1.31) | 0.007* |
| First visited HCF | DOTS center | 241.61(0.1) | | Ref. |  | Ref. |  |
|  | Non DOTS center | 250.28(0.1) | | 1.04(0.91,1.18) | 0.6 | 0.94(0.85,1.05) | 0.4 |
| TB diagnosed HCF | Public | 237.90(0.1) | | Ref. |  | Ref. |  |
|  | Private | 292.16(0.11) | | 1.23(1.02,1.48) | 0.03 | 1.07(0.93,1.23) | 0.4 |
| Number of HCF visited until Diagnosis ^b^ | | | | 1.40(1.34,1.47) | <0.001 | 1.09(1.05,1.14) | 0.005* |
| Hospitalized for treatment | Yes | 477.85(0.12) | | 2.0(1.46,2.76) | <0.001 | 1.97(1.56,2.48) |  |
|  |  |  |  |  |  |  |  |
|  | No | 237.81(0.1) | | Ref. |  | Ref. | <0.001* |

^a^ exponent to the power 10, ^b^ variable treated as continuous, ^c^ self treatment, used holy water, consult traditional healer, *statistically significant at p<0.05
